# Increased static dielectric constant in *ZnMnO* and *ZnCoO* thin films with bound magnetic polarons

**DOI:** 10.1038/s41598-020-63195-1

**Published:** 2020-04-21

**Authors:** Sahitya V. Vegesna, Vinayak J. Bhat, Danilo Bürger, Jan Dellith, Ilona Skorupa, Oliver G. Schmidt, Heidemarie Schmidt

**Affiliations:** 1 Leibniz Institute of Photonic Technology, 07745 Jena, Germany; 2 Institute for Solid State Physics, Friedrich Schiller University Jena, 07743 Jena, Germany; 3 Fraunhofer Institute for Electronic Nano Systems, Department Back-End of Line, 09126 Chemnitz, Germany; 4 Institute of Ion Beam Physics and Materials Research, Helmholtz-Research Center Dresden-Rossendorf, 01314 Dresden, Germany; 5 Institute for Integrative Nanosciences, Leibniz Institute for Solid State and Materials Research Dresden, 01069 Dresden, Germany; 6 Material Systems for Nanoelectronics, Technische Universität Chemnitz, 09126 Chemnitz, Germany

**Keywords:** Magnetic properties and materials, Spintronics, Semiconductors

## Abstract

A novel small signal equivalent circuit model is proposed in the inversion regime of metal/(*ZnO*, *ZnMnO*, and *ZnCoO*) semiconductor/*Si*_3_*N*_4_ insulator/p-Si semiconductor (MSIS) structures to describe the distinctive nonlinear frequency dependent capacitance (*C-F*) and conductance (*G-F*) behaviour in the frequency range from 50 Hz to 1 MHz. We modelled the fully depleted *ZnO* thin films to extract the static dielectric constant (*ε*_*r*_) of *ZnO*, *ZnMnO*, and *ZnCoO*. The extracted enhancement of static dielectric constant in magnetic n-type conducting *ZnCoO* (*ε*_*r*_ ≥ 13.0) and *ZnMnO* (*ε*_*r*_ ≥ 25.8) in comparison to unmagnetic ZnO (*ε*_*r*_ = 8.3–9.3) is related to the electrical polarizability of donor-type bound magnetic polarons (*BMP*) in the several hundred GHz range (120 GHz for *CdMnTe*). The formation of donor-BMP is enabled in n-type conducting, magnetic *ZnO* by the s-d exchange interaction between the electron spin of positively charged oxygen vacancies ${V}_{o}^{+}$ in the *BMP* center and the electron spins of substitutional Mn^2+^ and Co^2+^ ions in *ZnMnO* and *ZnCoO*, respectively. The *BMP* radius scales with the Bohr radius which is proportional to the static dielectric constant. Here we show how *BMP* overlap can be realized in magnetic *n-ZnO* by increasing its static dielectric constant and guide researchers in the field of transparent spintronics towards ferromagnetism in magnetic, *n-ZnO*.

## Introduction

The favourable electrical and optical properties of zinc oxide made it promising for applications in opto-electronics^1^, sensor technology^2^, UV light emitting diodes^3^, and photovoltaic devices. In the field of spintronics, special attention has been given to oxygen-deficient magnetic *ZnO* thin films with substitutional 3d transition metal ions^4–6^. Observed spontaneous magnetization has been related with the formation of stable Bound Magnetic polarons (*BMP*)^7^. The *BMP* concept was first introduced to explain metal-insulator transition in oxygen-deficient *EuO*^8^. *BMPs* are formed by the *s-d* exchange interactions between the electron spin of a singly charged oxygen vacancy ${V}_{o}^{+}$ in the center of the *BMP* and the electron spins of substitutional *3d* transition metal ions in a sphere with Bohr radius *r*_*B*_^9–11^. The Bohr radius is proportional to the static dielectric constant. Due to the *s-d* exchange interaction between the spin of singly charged oxygen vacancy ${V}_{o}^{+}$ and the spins of the *3d* transition metal ions in the sphere with Bohr radius *r*_*B*_, the spins of the *3d* transition metal ions the align in same direction and sum up to the collective spin of the *BMP*. For example, spontaneous magnetization due to collective spins of *BMPs* in *CdTe* with substitutional *Mn* ions was reported by Peter and Eucharista^12^. From magnetic *n-CdS*^13,14^ and *n-CdSe*^15,16^ there is abundant evidence that the electron localized at the impurity in the *BMP* center can induce sizable magnetization in its vicinity, often having magnetic moments exceeding 25 *μ*_*B*_^17^. Interestingly, so far the focus in the *BMP* research was more on the formation of *BMP* and not on the increase of the static dielectric constant in the dilute magnetic semiconductor in comparison to the semiconductor host without substitutional magnetic ions. For example, the static dielectric constant of *ZnO* amounts to 8.5–9.5^18–20^ and we have observed an increase of the static dielectric constant of *ZnCoO* up to 25.0 if 4 at.% *Co* is added^7^. Investigations of dielectric constant of *ZnCoO* powders modelled from measured shift in bandgap showed that it is not possible to achieve significant increase in dielectric constant. This may be due to the absence of singly ionised oxygen vacancies (${V}_{o}^{+}$) in *ZnCoO* powders enabling *s-d* exchange interaction and bound magnetic polaron formation which would enhance the static dielectric constant of *ZnCoO* powders. In this work we determine the magnetic species and concentration dependent static dielectric constant *ε*_*r*_ of two *ZnO* thin films and eight magnetic *ZnO* thin films with 2 at.% and 5 at.% substitutional *Co*^2+^ and *Mn*^2+^ ions from analysis of capacitive metal/*n-ZnO* semiconductor/*Si*_3_*N*_4_ insulator/*p-Si* semiconductor (*MSIS*) structures. The oxygen partial pressure during growth of the magnetic *n-ZnO* films by pulsed laser deposition (*PLD*) mainly determines the concentration of oxygen vacancies which are intrinsic donors and may form the center of *BMP* in magnetic *ZnO*. The intrinsic oxygen vacancy defects are donors that can be estimated from room temperature sheet resistance. This work proposes an approach to determine intrinsic defects from measured sheet resistance and volume of bound magnetic polaron which are the main ingredients that guide researchers towards ferromagnetism in transparent spintronics. The static dielectric constant has been modelled from the measured frequency dependent capacitance characteristics (*C-F*) of *MSIS* structures. The simpler metal insulator metal (*MIM*) structure for evaluation of static dielectric constant of magnetic, n-type conducting *ZnO* layers would be problematic for modelling frequency dependent capacitance data. This is because even nominally insulating *ZnO* thin films in *MIM* structures are leaky insulators and such MIM structures are not suitable for analysing non-linear frequency dependent impedance. And also, the analysis of current voltage (*IV*) and impedance (*CV*) data of Schottky diodes with completely depleted *ZnO* thin films have too many unknown implicit parameters to extract the static dielectric constant of the *ZnO* thin film in a Schottky diode from the *IV* and *CV* data. Schottky diodes with n-type conducting *Zn*_0.95_*Co*_0.05_*O* thin films have been investigated by Kasper *et al*.^7^. Kasper *et al*. used a static dielectric constant of *ε*_*r*_ = 25^21^. It was not possible to extract the static dielectric constant of *Zn*_0.95_*Co*_0.05_*O*. Therefore, we chose a *MSIS* heterostructure in order to extract the static dielectric constant of magnetic, n-type conducting *ZnO* layers.

## Results

**Figure 1:**
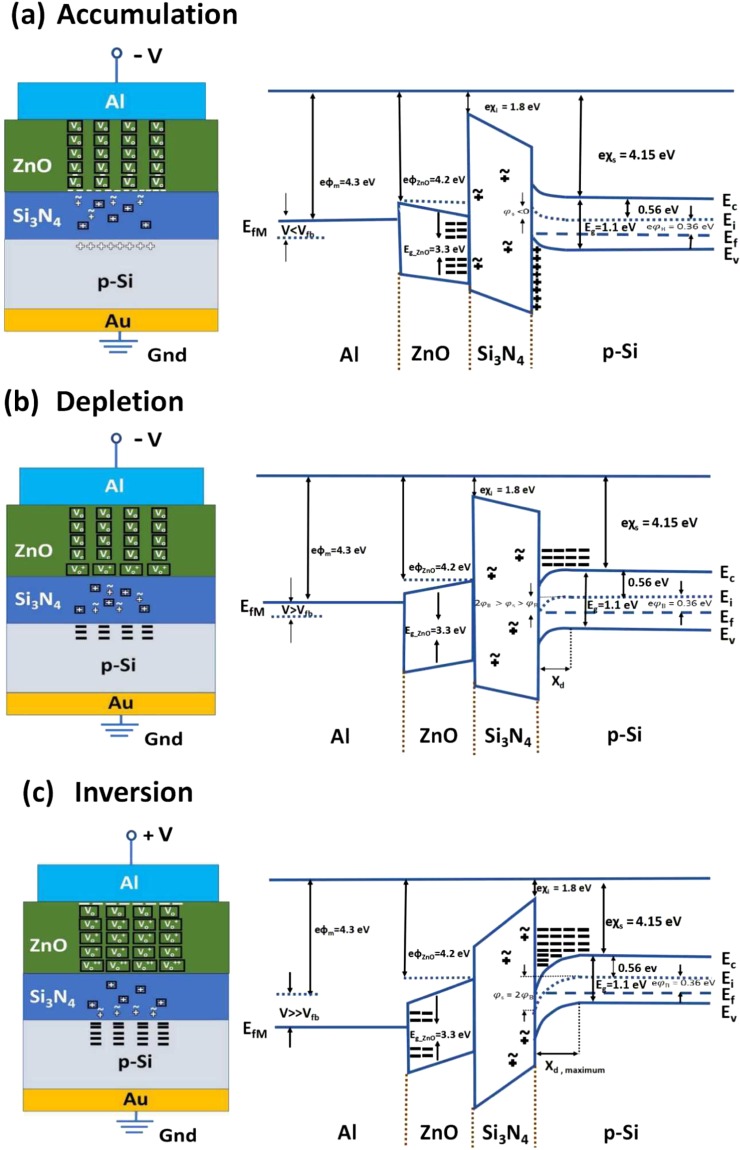
Schematic representation of charge distribution in the *n-ZnO* layer, the *Si*_3_*N*_4_ layer and the *p-Si* in the metal/*n-ZnO* semiconductor/*Si*_3_*N*_4_ insulator/*p-Si* semiconductor (MSIS) structure and corresponding band diagram in (**a**) accumulation (**b**) depletion and (**c)** inversion. There are mirror charges on Al top electrode to compensate the charges in *p-Si* accumulated at the interface *Si*_3_*N*_4_/*p-Si*. There are singly ionized oxygen vacancies in accumulation and depletion and single and double ionized oxygen vacancies in inversion in Z*nO*. The majority charge carriers are accumulated at the opposite interface of the Z*nO* layer. *Si*_3_*N*_4_ contains both mobile (~) and fixed (▫) positively charged impurities. The existence of the positive impurity charges are expected from the shift of the flat-band voltage towards more negative biases in the negative bias range. Due to the thickness (~110 nm) of the n-type Z*nO* thin film, only fully depleted or fully accumulated regime band diagram is shown in the figure. Work function of Φ_*M*_ for aluminium metal is 4.3 eV, electron affinity of Z*nO χ*_*ZnO*_ is 4.2 eV, electron affinity of *Si*_3_*N*_4_ *χ*_*i*_ is 1.8 eV and electron affinity of p-Si *χ*_*s*_ is 4.15 eV. Band gap of ZnO ${{\rm{E}}}_{g}^{ZnO}$ is 3.3 eV, band gap of p-Si ${{\rm{E}}}_{g}^{Si}$ is 1.1 eV and bulk potential *φ*_*b*_ of p-Si is 0.36 eV.

Oxygen vacancies in *n-ZnO* are intrinsic donors and increase the concentration of the electron majority charge carriers *n*. If the carrier concentration *n* is small, the *ZnO* thin films in the metal/*n-ZnO* semiconductor/*Si*_3_*N*_4_ insulator/*p-Si* semiconductor *MSIS* structures are insulating. With decreasing *n* the carrier mobility *μ* increases and influences the dc transport properties of the *ZnO* in the (*MSIS*) structures. The *ZnO*, *ZnCoO*, and *ZnMnO* thin films have been grown by *PLD* on insulator-semiconductor (*Si*_3_*N*_4_*/p-Si*) *MIS* structures for investigating the static dielectric constant of the magnetic *ZnO* thin films (Fig. 1). In the following we show how measured impedance has been modelled and how the extracted capacitance of the magnetic *ZnO* thin films has been used to extract the static dielectric constant of magnetic *ZnO* in dependence on the species and concentration of magnetic ions. The polarity and strength of the applied bias on the *Al*/*ZnO* interface determines the ionization of donor oxygen vacancies (${V}_{o}$) (Fig. 1(a–c)). The mobile defects in *Si*_3_*N*_4_ are redistributed in *Si*_3_*N*_4_ under a bias applied to the *MSIS* structure, namely with large negative applied bias in accumulation towards the ZnO/*Si*_3_*N*_4_ interface (Fig. 1(a)) and for a large positive applied bias inversion towards the Si_3_N_4_/p-Si interface (Fig. 1(c)). The flat band voltage lies in the negative bias range (Fig. S3 in supplementary) for both ramping directions, namely from accumulation (Fig. 1a) to inversion (Fig. 1c) and from inversion to accumulation. This indicates the presence of positive charge defects in *Si*_3_*N*_4_ (Fig. S3 in supplementary). *Si*_3_*N*_4_ contains both mobile (~) and fixed (▫) positive charge defects. The presence of fixed impurities and mobile positive charge defects in insulating *Si*_3_*N*_4_ can be recognized from shift flat band voltage and midgap voltage of conductance and capacitance hysteresis measurements, respectively (Fig. S3). First the distribution of mobile defects in *Si*_3_*N*_4_ is changed when the dc bias is ramped from +10 V to −15 V (accumulation in Fig. 1(a)) or when the dc bias is ramped from −15 V to +10 V (depletion-inversion in Fig. 1(b,c)). The positive fixed and mobile charge defects in the insulating Si_3_N_4_ layer cause a shift of the flat band voltage to larger negative bias. The mobility of the mobile defects in *Si*_3_*N*_4_ depends on the *PLD* growth temperature during deposition of the n-type semiconductor on the insulator *Si*_3_*N*_4_, namely 550 °C for the deposition of *ZnO* in this work and 380 °C for the deposition of *BiFeO*_3_ in a previous work^22^. It has been reported that the threshold temperature for the formation of defects in *Si*_3_*N*_4_ lies at circa 500 °C^23^.

**Figure 2:**
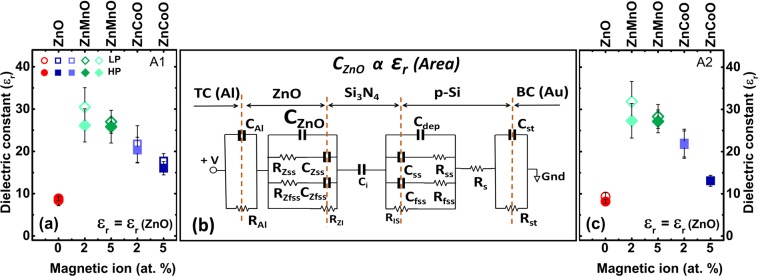
Modelled static dielectric constant of *ZnO* (°), *ZnCoO* (▫), and *ZnMnO* (◊) for top contact area (**a**) A1 [5.026 × 10^−7^ m^2^] and (**c**) A2 [2.827 × 10^−7^ m^2^]. The variation of the static dielectric constant which is extracted from C_*ZnO*_ used for modelling [1 ± (Δ/2)] × C_*ZnO*_ (s.a. error of C_*ZnO*_ in Table 1) is indicated as an error bar. Samples grown under low oxygen partial pressure (LP) with 6.50 × 10^−3^ mbar and under high oxygen partial pressure (HP) samples with 3.91 × 10^−2^ mbar are shown in open and closed symbols respectively. (**b**) Equivalent circuit model for *Al/ZnO/Si*_3_*N*_4_*/p-Si/Au MSIS* structure at inversion regime (Fig. 1(c)).

The small signal analysis of *Al*/*n-ZnO* semiconductor/*Si*_3_*N*_4_ insulator/*p-Si* semiconductor structures was performed for obtaining the static dielectric constant of completely depleted *ZnO*, *ZnCoO*, and *ZnMnO* thin films (Fig. 1(c)). The *MSIS* equivalent circuit model in strong inversion is shown in Fig. 2(b)) and accounts for all *RC* elements in the interfaces and layers of the *MSIS* structure. The equivalent circuit model describes the measured nonlinear behaviour of the frequency dependent capacitance (*C-F*) and conductance (*G-F*) curve (Fig. S5 in supplementary) of samples grown under different oxygen partial pressures 6.5 × 10^−3^ mbar (*LP*), 3.91 × 10^−2^ mbar (*HP*) for two top contact areas *A1* (5.026 × 10^−7^ m^2^) and *A2* (2.827 × 10^−7^ m^2^).

The equivalent circuit model describes the impedance characteristics of each region in the *MSIS* structure that includes each material and the interface regions between the materials. The small signal impedance of *MIS* and of *MSIS* structures is analyzed in strong inversion (*s.a*. supplementary). An equivalent circuit model describing the frequency dependent capacitance (*C-F*) and conductance (*G-F*) of the reference structure, namely of *Al/Si*_3_*N*_4_*/p-Si/Au* metal/insulator/semiconductor (*MIS*) structures, is presented in ref. ^24^. In this work we also extended the MOS equivalent circuit model to describe voltage dependent impedance (*C-V* and G*-V*))^24^ to the *MSIS* equivalent circuit model with a *n-ZnO* semiconductor layer (Fig. 2(b)) (*s.a*. supplementary). The modelled parameters of the *MIS* structure (reference samples) have been used as an estimate for the corresponding parameters of the *MSIS* structures (*s.a*. S3.1). The modelling of small signal impedance of the *MSIS* structure always starts in the high frequency range where the leaky *Si*_3_*N*_4_ does not dominate frequency dependent small signal impedance (*s.a*. S3.2). Afterwards the small signal impedance has been modelled in the whole frequency range (*s.a*. S3.3). C_*ZnO*_ is the parameter which is finally used to extract the static dielectric constant of the *ZnO* layer in the *MSIS* structures.

The equivalent circuit model of the *MSIS* structure is given in Fig. 2(b). It describes the impedance characteristics of each layer in the *MSIS* structure and the interface regions between each layer. The capacitor *C*_*i*_ represents the *Si*_3_*N*_4_ capacitance. The *p-Si* region consists of *p-Si* depletion capacitance *C*_*dep*_ in series with the *Si*_3_*N*_4_ capacitor. The sharp termination of *p-Si* at the *Si*_3_*N*_4_*/p-Si* interface causes formation of surface states in *p-Si*. Those surface states are occupied during strong inversion^24^. The *MSIS* equivalent circuit model accounts for slow and fast surface states with capacitance/resistance *C*_*ss*_*/R*_*ss*_ and *C*_*fss*_*/R*_*fss*_, respectively, in parallel to the *p-Si* depletion capacitance *C*_*dep*_. The series resistance *R*_*S*_ includes resistances from undepleted *p-Si* in series with top electrode and bottom electrode. The bottom contact capacitance *C*_*st*_ in parallel with the resistor *R*_*st*_ in the circuit model emulates the Schottky junction between the bottom gold contact and semiconductor. The barrier height calculated from the modelled capacitance of bottom contact agrees with the calculation of barrier height from difference in work function of gold (4.8 eV)^25^ and work function of *Si* (5.07 eV)^26^. The sharp interface between *ZnO* and *Si*_3_*N*_4_ causes the formation of surface states in *ZnO* at the *ZnO/Si*_3_*N*_4_ interface. The *MSIS* equivalent circuit model (Fig. 2(b)) also accounts for the slow and fast surface states in *ZnO* with capacitance/resistance *C*_*Zss*_*/R*_*Zss*_ and *C*_*Zfss*_*/R*_*Zfss*_ in parallel with the depletion capacitance C_*ZnO*_ in *ZnO*, respectively. Also, charges at the interface of top contact aluminium (*Al*) and *ZnO* are taken into account with capacitance *C*_*Al*_ in parallel with the resistance *R*_*Al*_. Additional resistive elements *R*_*ZI*_ and *R*_*IS*_ (*R*_*ZI*_ = *R*_*IS*_) which describe the conductivity changes in the defective *Si*_3_*N*_4_ at the Z*nO/Si*_3_*N*_4_ and *Si*_3_*N*_4_*/Si* interfaces, respectively, have been incorporated into the *MSIS* equivalent circuit model to describe the defects in the *Si*_3_*N*_4_ (S3.4). In Fig. 2, dotted vertical lines indicate the interface between each layer. We show arrows at the interface position of *ZnO/Si*_3_*N*_4_ and *Si*_3_*N*_4_*/ZnO* to sketch that *R*_*ZI*_ and *R*_*IS*_ are finite and belong to the leaky *Si*_3_*N*_4_ dielectric. We see a frequency dependent capacitance for *Si*_3_*N*_4_ in small signal ac analysis. Also, a voltage dependent dc conduction is seen in leaky *Si*_3_*N*_4_. Therefore, *Si*_3_*N*_4_ can be considered as a broken ac channel with same dc conduction and for small signal equivalent circuit. Analytically we considered a capacitor with reduction in effective thickness described by Beaumont and Jacobs model^27^. Because ac conduction does not go through the *Si*_3_*N*_4_ at all frequencies and because of charge neutrality, the resistance change due to accumulation of charges at the interface *ZnO/Si*_3_*N*_4_ (*R*_*ZI*_) and at the interface *Si*_3_*N*_4_*/p-Si* (*R*_*IS*_) the corresponding resistance change is the same, i.e. *R*_*ZI*_ = *R*_*IS*_.

## Discussion

The dielectric constant of the *ZnO* layer in the *MSIS* structure has been determined from the modelled *C*_*ZnO*_ (Fig. 2(b)) using the area of the *Al* top contacts and the *ZnO* thickness from *SEM* measurements (Table S1 in supplementary). The static dielectric constant *ε*_*r*_ (Table 1) calculated for *ZnO*, *ZnCoO*, and *ZnMnO* grown at 6.50 × 10^−3^ mbar (*LP*), 3.91 × 10^−2^ mbar (*HP*) oxygen partial pressure is plotted in Fig. 2(a) for contact area *A1* and in Fig. 2(c) for contact area *A2* (*A1* = 5.026 × 10^−7^ m^2^ and *A2* = 2.827 × 10^−7^ m^2^). The modelled static dielectric constant of *ZnO* ranges between 8.2 and 9.3 and is in good agreement with literature values in the range between 8.5 and 9.5. A strongly increased static dielectric constant has been deduced from *C*_*ZnO*_ of *MSIS* structures with *ZnCoO* and *ZnMnO* thin films. We also see a slight increase of dielectric constant for *ZnO_LP* and *ZnO_HP* in comparison to bulk *ZnO*. However, it is not proven so far that the observed increase of dielectric constant in *ZnO* can be related with magnetism in *ZnO*, e.g. with magnetism due to the formation of bound magnetic polarons (*BMPs*). One could speculate that for *ZnO_LP* which has been grown at low oxygen partial pressure and which has a larger concentration of intrinsic donors, more donors are available as centres for *BMPs*. One possible type of ferromagnetic s-d exchange interaction in pure *ZnO* is the *s-d* exchange interaction between *3d* electrons of *Zn* ions and electron spin of oxygen vacancies (*Vo*^+^). Therefore, we expect an increased volume of bound magnetic polarons (Eq. (1)) in magnetic *ZnO* in comparison to unmagnetic *ZnO*.

**Table 1:** Modelled static dielectric constant of the *ZnO* thin films for *ZnO_LP*, *ZnO_HP*, *Zn*_1−*x*_*Co*_*x*_*O_LP*, *Zn*_1−*x*_*Co*_*x*_*O_HP*, *Zn*_1−*x*_*Mn*_*x*_*O_LP*, and *Zn*_1−*x*_*Mn*_*x*_*O_HP* from modelled capacitance (*C*_*ZnO*_) and measured *SEM* thickness (*s.a*. Supplementary Table S1).

Sample	Conductivity of ZnO	Contact	Thickness of ZnO (nm)	Modelled capacitance (mF/m^2^)	Dielectric constant
ZnO_LP	moderate	A1	093.0	0.80 ± 0.12	08.39 ± 1.25
		A2	093.0	0.89 ± 0.08	09.34 ± 0.93
ZnO_HP	insulating	A1	103.4	0.76 ± 0.04	08.87 ± 0.53
		A2	103.4	0.70 ± 0.04	08.17 ± 0.48
Zn_0.95_Co_0.05_O_LP	insulating	A1	120.6	1.30 ± 0.13	17.71 ± 1.77
		A2	120.6	0.96 ± 0.09	13.07 ± 1.30
Zn_0.95_Co_0.05_O_HP	insulating	A1	118.3	1.12 ± 0.11	16.03 ± 1.60
		A2	118.3	0.98 ± 0.09	13.09 ± 1.30
Zn_0.98_Co_0.02_O_LP	low	A1	120.3	1.60 ± 0.32	21.74 ± 4.34
		A2	120.3	1.62 ± 0.24	22.01 ± 3.30
Zn_0.98_Co_0.02_O_HP	low	A1	118.3	1.52 ± 0.23	20.31 ± 3.04
		A2	118.3	1.62 ± 0.24	21.64 ± 3.24
Zn_0.95_Mn_0.05_O_LP	insulating	A1	116.6	2.05 ± 0.20	27.00 ± 2.70
		A2	116.6	2.15 ± 0.21	28.31 ± 2.83
Zn_0.95_Mn_0.05_O_HP	insulating	A1	117.3	1.95 ± 0.30	25.83 ± 3.87
		A2	117.3	2.05 ± 0.20	27.16 ± 2.71
Zn_0.98_Mn_0.02_O_LP	moderate	A1	120.0	2.25 ± 0.35	30.49 ± 4.57
		A2	120.0	2.35 ± 0.35	31.84 ± 4.77
Zn_0.98_Mn_0.02_O_HP	insulating	A1	101.5	2.28 ± 0.34	26.14 ± 3.92
		A2	101.5	2.38 ± 0.36	27.28 ± 4.09

The variation of *C*_*ZnO*_ is indicated as an error in Table 1 and the extracted static dielectric constant has an error bar corresponding to the variation of the dielectric constant which is extracted from *C*_*ZnO*_ used for modelling [1 ± (Δ/2)] × C_*ZnO*_ in the frequency range from 10^3^ to 6 × 10^4^ Hz where the capacitance of the whole *MSIS* structure is most sensitively depending on *C*_*ZnO*_. Conductivity of *ZnO* thin films have been measured separately with the Hall measurement in van der Pauw geometry. Sheet resistance of *ZnO_LP* is 1.91 × 10^7^ ohm/▫, of *Zn*_0.98_*Co*_0.02_*O_LP* is 4.55 × 10^7^ ohm/▫, of *Zn*_0.98_*Co*_0.02_*O_HP* is 1.56 × 10^7^ ohm/▫, and *Zn*_0.98_*Mn*_0.02_*O_LP* is 0.09 × 10^−7^ ohm/▫. The free carrier concentration of the *ZnO_LP* and *Zn*_0.98_*Mn*_0.02_*O_LP* is in the range of 10^14^ cm^−3^. The free carrier concentration is expected to smaller than the donor concentration because *ZnO* thin films in the in strong inversion of *MSIS* structures are completely depleted.

The resistance of the *ZnO* has been measured and the transport properties are classified^28,29^ by ranges of resistance in Table 1. Insulating *ZnO* thin films have lower *ε*_*r*_ while low conducting *ZnO* and moderate conducting *ZnO* thin films have higher *ε*_*r*_ which is an indication of the dielectric constant dependence on donor concentration. Here the donors are intrinsic donors formed in *ZnO* by oxygen vacancies (${V}_{o}$) whose concentration depends on the oxygen partial pressure during *PLD* growth of *ZnO*. One might expect smaller dielectric constant in higher pressure (*HP*) samples in comparison to lower pressure (*LP*) samples, because electrically polarizable *BMP* represent a collective spin of *3d* spins of *Mn*^2+^ in *ZnMnO* and of *Co*^2+^ spins in *ZnCoO* which is mediated by *s-d* exchange interaction between *3d* wavefunction of *3d* spins and *s* wavefunction of the electron spin of *Vo*^+^ in the centre of the bound magnetic polaron^30^. More *BMPs* are expected for a larger number of oxygen vacancies in lower pressure samples.

There exist three types of known native donors in *ZnO* oxide, i.e., *O* vacancies (*Vo*), *Zn* interstitials (*I*_*Zn*_), and H related defects (*H*_*i*_)^31^ which play crucial roles in determining the transport and optical properties of zinc oxide. We investigated the species of shallow donors in *ZnO* thin films grown by pulsed laser deposition by assuming two different donors with two thermal activation energies in the *ZnO*. For example, in our previous work Vegesna *et al*.^28^ the existence of two different donors could (${{\rm{E}}}_{a}^{1}$ = 1.54 meV and ${{\rm{E}}}_{a}^{2}$ = 82.75 meV) be proven by modeling the temperature dependent free carrier concentration. This thermal activation energy hints towards hydrogen related defects and zinc interstitials. Because the thermal activation energy of oxygen vacancies amounts to 300 meV Hofmann *et al*.^32^, it is not possible to prove existence of oxygen vacancies in *ZnO* by temperature dependent transport measurements. Hoffman *et al*. used photoluminescence measurements and related the green emission from *ZnO* with the existence of oxygen vacancies. In a recent work Liu *et al*.^33^ showed that oxygen vacancies are the dominant defects in n-type conducting *ZnO* using oxygen isotope diffusion which depends on the concentration of oxygen vacancies. Here we focus on native point defects providing a single electron spin for the formation of *BMP* in magnetic, intrinsically n-type conducting *ZnO*. The only native donor in *n-ZnO* carrying a single electron spin is the O vacancy (${V}_{o}^{+}$). Zinc interstitials occur exclusively in the 2^+^ charge state, i.e., ${I}_{Zn}^{++}$^34^. Therefore, formation of bound magnetic polarons with ${I}_{Zn}^{++}$ (no electron, S = 0), *I*_*Zn*_ (paired electrons, S = 0) and ${H}_{i}^{+}$ (no electrons, S = 0) is not possible. Only singly ionised oxygen vacancy (${V}_{o}^{+}$) (single electron, S = 1/2) can form the center of *BMP*. ${V}_{o}$ (paired electrons, S = 0) and ${V}_{o}^{2+}$ (no electron, S = 0) with zero-valued electron spin cannot be the center of the a donor-*BMP*^35^.

The spin interaction volume in *BMP* constitutes^30^ represent a collective spin of *3d* spins of *Mn*^2+^ and *Co*^2+^ which is mediated by *s-d* exchange interaction between *3d* wavefunction of *3d* spins and *s* wavefunction of the spin of ${V}_{o}^{+}$ in the center of the bound magnetic polaron. The volume of bound magnetic polaron defined by the Bohr radius is proportional to the static dielectric constant. The Bohr radius can be calculated using following equation

1 $${r}_{b}=\frac{4\pi {\varepsilon }_{0}{\varepsilon }_{r}\hslash }{m{e}^{2}},$$

where *ε*_0_ is permittivity of free space, $\hslash$ is reduced Planck's constant, *ε*_*r*_ is static dielectric constant, *m* is effective mass (0.24*m*_0_)^36^ and *e* is elementary charge.

The bound magnetic polaron (*BMP*) in *ZnCoO* and in *ZnMnO* has a huge collective spin, if many *3d* ions lie in the volume of the bound magnetic polaron. The larger the number of *3d* ions in the *BMP* volume, the more spins of *3d* ions can be aligned in parallel by the *s-d* exchange between the spin of the oxygen vacancy (${V}_{o}^{+}$) in the center of the *BMP* and the spins of the *3d* ions in the *BMP* volume within the Bohr radius^37^. The *BMP* will increase the polarizability of magnetic *ZnO*.

In our work, we have extracted the static dielectric constant from frequency dependent impedance data measured on *ZnO* coated *MSIS* structures. The model does not capture frequency dependence of the dielectric constant of *ZnO*. In the measured frequency region up to 1 MHz the dielectric constant of *ZnO* are expected to be constant. Therefore, a time dependent switching characteristics of static dielectric constant in *ZnO* can only be studied if the switching is non-volatile. For example, the model could possibly be used to investigate the dynamics of spin alignment in *BMPs* in magnetic, *n-ZnO* if single magnetic field pulses of different lengths are applied before the measurement of impedance data in dependence on the magnetic field pulse length. Before applying subsequent magnetic field pulse and before measuring the resulting frequency dependent impedance data, the spin alignment in the *BMP* has to be destroyed, e.g. by an ac magnetic field. We expect that the dynamics of the spin alignment in *BMPs* will depend on the volume and on the material dependent ferromagnetic s-d exchange parameter. A direct measurement of the spin dynamics in *BMP* would be possible if the frequency dependence of the dielectric constant could be measured in the several hundred GHz frequency range, e.g. by microwave measurements.

**Figure 3:**
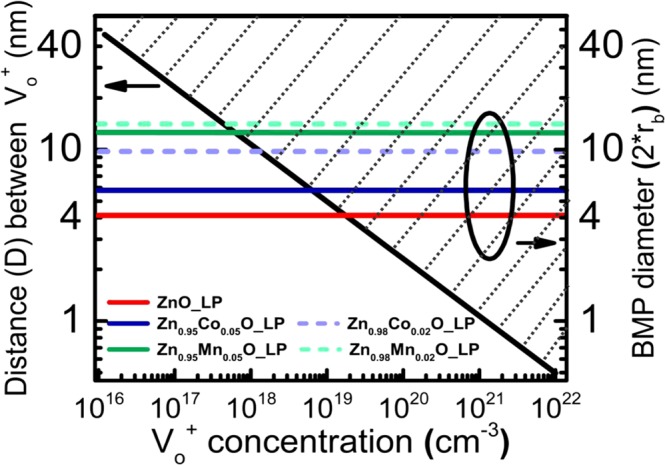
Calculated distance between the homogeneously distributed oxygen vacancies (black line) for *ZnO* in dependence on oxygen vacancy concentration (${{\rm{V}}}_{o}^{+}$) in logarithmic scale. Calculated sample dependent bound magnetic polaron (BMP) diameter represented in the same range of ${{\rm{V}}}_{o}^{+}$. Oxygen vacancies overlap in the dotted area for ${{\rm{V}}}_{o}^{+}$ concentrations larger than the ${{\rm{V}}}_{o}^{+}$ concentration (intersection of colored lines and black line) where *BMP* diameter and distance between ${{\rm{V}}}_{o}^{+}$ are equal.

In the following we discuss possible percolation of *BMP* in *ZnO* with dependence on the static dielectric constant and the concentration of oxygen vacancies. Coey and Venkatesan^30^ estimated the concentration of defects in *ZnO* for polaron percolation based on a static dielectric constant of *ZnO* of (*ε*_*r*_) and Bohr radius (*r*_*H*_). A threshold concentration of defects in*ZnO* of 4 × 10^19^ cm^−3^ has been obtained for *ε*_*r*_ = 4.0 and *r*_*H*_ = 0.76 nm from (${n}_{\square }^{crit}$)^1/3^ *r*_*H*_ ≈ 0.26^38^, where ${n}_{\square }^{crit}$ is the critical defect concentration for delocalization of the impurity band states. In Fig. 3 we show the calculated *BMP* diameter in *ZnO* thin films with different static dielectric constants (*ε*_*r*_(A1) = 8.39 (*ZnO_LP*), 17.71 (*Zn*_0.95_*Co*_0.05_*O_LP*), 21.74 (*Zn*_0.95_*Co*_0.02_*O_LP*), 27.00 (*Zn*_0.95_*Mn*_0.05_*O_LP*), and 30.49 (*Zn*_0.95_*Mn*_0.02_*O_LP)*) and with the density of oxygen vacancies ranging from 10^16^ cm^−3^ to 10^22^ cm^−3^. For simplicity, for the determination of the distance between oxygen vacancies we have considered a homogeneous oxygen vacancy distribution. The diagonal black solid line gives the distance between two oxygen vacancies in dependence on concentration of oxygen vacancies. If the distance between the oxygen vacancies is smaller than the diameter of *BMP*, *BMPs* coalesce and overlap. Such overlap of bound magnetic polarons possibly induces ferromagnetism in magnetic *ZnO* at room temperature^39,40^ if the orientation of the electron spin of the oxygen vacancy in the center of *BMP* is stable and not continuously changing due to hopping transport of free carriers via oxygen vacancies.

We describe the frequency dependent capacitance (*C-F*) behaviour of the *Al*/*n-ZnO* semiconductor/*Si*_3_*N*_4_ insulator/*p-Si* semiconductor *MSIS* structure with an equivalent circuit model in strong inversion regime where each layer and interface has been described. Static dielectric constant of *ZnO* has been extracted from modelled capacitance of the *ZnO* layer. The dielectric constant of *ZnO* lies in the expected range from 8.1 to 9.3. We observed strongly increased static dielectric constant in magnetic *ZnO* in dependence on the concentration of magnetic ions and on the concentration of oxygen vacancies. The dielectric constant in *ZnMnO* with 5 at. % *Mn* is 28.3 and with 2 at. % *Mn* is 31.8. The dielectric constant in *ZnCoO* with 5 at. % *Co* is 17.7 and with 2 at. % *Co* is 22.0. The ferromagnetic *s-d* exchange interaction between electron spin of donors (${V}_{o}^{+}$) in the center of the bound magnetic poloron (*BMP*) and the electron spin of substitutional magnetic ions is partially superimposed by the anti-ferromagnetic coupling between nearest neighbours substitutional magnetic ions. With increasing concentration of substitutional magnetic ions it is expected that the anti-ferromagnetic coupling which excludes ferromagnetic *s-d* coupling increases and weakens the formation of *BMPs*. This is the possible reason why we see a larger static dielectric constant in magnetic *ZnO* with 2 at. % substitutional magnetic ions in comparison to magnetic *ZnO* with 5 at. % substitutional magnetic ions. The observed trend is in agreement with the observations from Franco *et al*.^41^ on powdered *ZnCoO* who observed a maximum of static dielectric constant in powdered *ZnCoO* around 2 at. % *Co*. We related the increased static dielectric constant in magnetic *ZnO* with the formation of partially overlapping bound magnetic polarons and their contribution to the electrical polarizability of magnetic *ZnO*.

**Figure 4:**
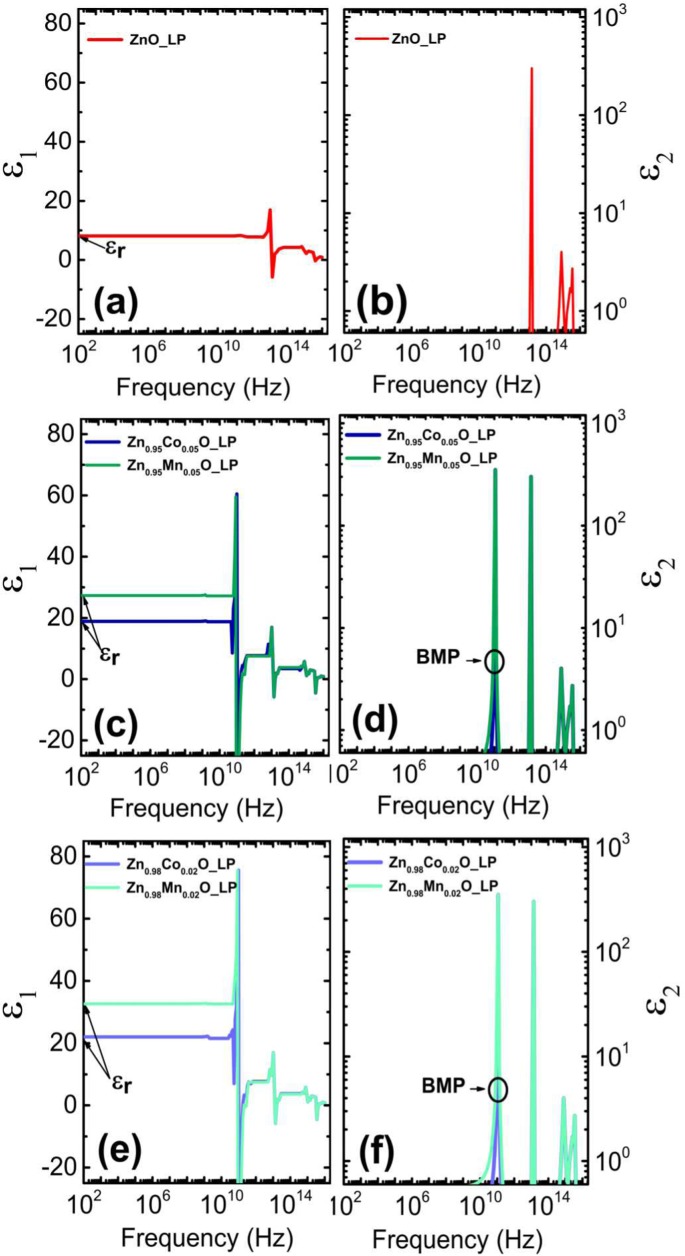
Real part (*ε*_1_) of dielectric constant for (**a**) *ZnO*, (**c**) *Zn*_0.95_*Co*_0.05_*O*, *Zn*_0.95_*Mn*_0.05_*O*, and (**e**) *Zn*_0.98_*Co*_0.02_*O*, *Zn*_0.98_*Mn*_0.02_*O* has been estimated by applying Kramers-Kronig tranformation to imaginary part (*ε*_2_) of dielectric constant for (**b**) *ZnO*, (**d**) *Zn*_0.95_*Co*_0.05_*O*, *Zn*_0.95_*Mn*_0.05_*O*, and (**f**) *Zn*_0.98_*Co*_0.02_*O*, *Zn*_0.98_*Mn*_0.02_*O*, respectively. The electronic^44^ and phonon^43^ contribution to *ε*_2_ has been taken from literature^43,44^. An additional contribution to *ε*_2_ due to *BMP* at 120 GHz has been assumed in such a way that *ε*_1_ agrees with modelled *ε*_*r*_.

Finally, we estimated the contribution of the *BMP* in *ZnO* to the polarizability of *ZnO*. The resonance of *BMP* typically lies in the several hundred GHz range. Here we chose the same resonance of *BMP* in magnetic *ZnO* as shown for the magnetic semiconductor *CdMnTe* where an additional absorption due to *BMP* has been observed at 120 GHz by Raman shift measurements (4 cm^−1^)^42^. We assumed an additional polarizability of magnetic *ZnO* due to *BMP* and added this to the modelled imaginary part (*ε*_2_) of the dielectric constant (Fig. 4(b,d,f)).

2 $${\varepsilon }_{2}(x)={\varepsilon }_{2}^{BMP}(x)+{\varepsilon }_{2}^{Phonon}(x)+{\varepsilon }_{2}^{Electronic}(x)$$

where ${\varepsilon }_{2}^{BMP}$ is the contribution due to *BMP*, ${\varepsilon }_{2}^{Phonon}$ is the contribution due to phonons in *ZnO*^43^ and where ${\varepsilon }_{2}^{Electronic}$ is the contribution due to electronic transitions in *ZnO*^44^. ${\varepsilon }_{2}^{BMP}$ has been described with a Lorentz oscillator model as follows:

3 $${\varepsilon }_{2}=1+{N}_{peak}\frac{\Gamma \omega }{{({\omega }_{o}^{2}-{\omega }^{2})}^{2}+{\Gamma }^{2}{\omega }^{2}}$$

where *ω*_*o*_ is the *BMP* peak position (*ω*_*o*_ = 120 GHz), *N*_*peak*_ is the peak strength and Γ is the *FWHM*. We calculated the real part (*ε*_1_) of the dielectric constant (Fig. 4(a)) using Kramers-Kronig relation (Eq. (4)) for *ZnO* with the electronic^44^ and phonon^43^ contribution to *ε*_2_. Additionally, the *FWHM* of a Lorentz oscillator with a fixed peak strength(*N*_*peak*_ = 350) and fixed peak position has been varied to change the contribution from ${\varepsilon }_{2}^{BMP}$ to *ε*_2_ in Fig. 4(d,f)) and derived *ε*_1_ of magnetic *ZnO* in Fig. 4(c,e), respectively, using Kramers-Kronig relation (Eq. (4)) ${\varepsilon }_{2}^{BMP}$ as long as static dielectric constant *ε*_1_ from Eq. (4) was the same as the modelled static dielectric constant from impedance measurements (*ε*_*r*_).

4 $${\varepsilon }_{1}(\omega )={\varepsilon }_{\infty }+\frac{2}{\Pi }{\int }_{0}^{\infty }\frac{x\cdot {\varepsilon }_{2}(x)}{{x}^{2}-{\omega }^{2}}$$

Estimated *FWHM* for *Zn*_0.95_*Co*_0.05_*O* is Γ = 0.7 GHz, *Zn*_0.95_*Mn*_0.05_*O* is Γ = 4.1 GHz, *Zn*_0.98_*Co*_0.02_*O* is Γ = 0.8 GHz, and *Zn*_0.98_*Mn*_0.02_*O* is Γ = 6.1 GHz.

We expect that the dielectric constant peak position can be tuned via the material dependent ferromagnetic *s-d* exchange parameter. Here we rather focused on the amplitude of the additional absorption ${\varepsilon }_{2}^{BMP}$ in the several hundred GHz range. We expect that the amplitude can be tuned via via the volume of the *BMP*. Dielectric constant shown in Fig. 4 represents the dielectric constant of magnetic *ZnO* layer in the *MSIS* structure. So far, we have not directly investigated the properties of *BMPs* in the several hundred GHz range.

*ZnO* coated *Si*_3_*N*_4_/*p-Si* metal insulator semiconductor (*MSIS*) structures with nominal concentration of 2 at.% and 5 at.% *Co*^2+^, *Mn*^2+^ ions at 6.50 × 10^−3^ mbar, 3.91 × 10^−2^ mbar oxygen partial pressure are grown by pulse layer deposition (*PLD*). Voltage dependent capacitance (*C-V*) and frequency dependent capacitance (*C-F*) characteristics have been measured. Thickness of *ZnO* layer and Si_3_N_4_ is obtained from secondary electron microscopy (*SEM*) cross section images. Measured *C-F* characteristics at strong inversion regime of *ZnO* coated *MSIS* structure shows, nonlinear behaviour of the capacitance. To describe the nonlinear behaviour of the *C-F* characteristics we proposed an equivalent circuit model at strong inversion regime. The *RC* equivalent circuit model gives the description of each region of *Al/ZnO/Si*_3_*N*_4_*/p-Si/Au MIS* structure such as metal, insulator, semiconductor including interface region between materials. Dielectric constant is obtained from modelled *ZnO* capacitance value and with the thickness of *ZnO* from *SEM* measurements. Dielectric constant for *ZnO* is obtained in the expected range *ε*_*r*_ = 8.17–9.34. We determined the static dielectric constant in magnetic, n-type conducting *ZnO* thin films with different *Co* and *Mn* concentration. With 2 at. % it is 31.84 and for 5 at.% *Mn* sample dielectric constant is 28.31 and for 2 at.% *Co* samples dielectric constant is 22.31 and for 5 at.% *Co* sample it is 17.71. We attribute the increase of the static dielectric constant to the contribution of bound magnetic polarons to the electrical polarization of magnetic, n-type conducting *ZnO*.

With increase in oxygen vacancies at the surface, bound magnetic polaron formed with oxygen vacancy as nucleus can overlap and provide ferromagnetic behaviour at room temperature^45^ Davies *et al*.^46^ and Kaspar *et al*.^7^ suggest that ferromagnetic features from bound magnetic polaron can be used in developing magnetic sensors, non-volatile memories in spintronics devices which are potentially expected to be energy-efficient devices. Application of *BFO* coated *Si*_3_*N*_4_ MIS structure as a photocapacitive detector has been studied by You *et al*.^22^. Because *ZnO* is transparent and because the *ZnO* coated *Si*_3_*N*_4_ *MIS* structure shows similar capacitance behaviour as the *BFO* coated *Si*_3_*N*_4_ *MIS* structure, the *ZnO* coated *Si*_3_*N*_4_ *MIS* structure is expected to reveal similar photocapacitive functionality as the *BFO* coated *Si*_3_*N*_4_ *MIS* structure to detect intensity and color of visible light by impedance measurements. In addition, we suggest to use the *ZnO* coated *Si*_3_*N*_4_ *MIS* capacitor as magneto-capacitive detector where the presence of a magnetic field can be detected via the increase of static dielectric constant due to the formation of *BMPs* with aligned spins of magnetic ions.

We propose to study change of static dielectric constant in magnetic transparent conducting oxides (*TCO*)^47,48^ by preparing metal/*n-TCO*/insulator/*p-Si MSIS* structures and by measuring and modelling the impedance in strong inversion. It is expected that also other magnetic n-type conducting *TCOs* reveal an increase of static dielectric constant due to the formation of bound magnetic polarons and due to the contribution of *BMP* to the polarizability of magnetic *TCOs*. Bound magnetic polarons strongly influence transport, magnetization and magnetooptical properties in magnetic semiconductors within the confined volume of *BMPs*. For example, ferromagnetic behaviour in magnetic *ZnO* at room temperature can be related with *BMP*^45,49^ and it has been suggested that ferromagnetic behavior related with *BMP* formation in magnetic n-type conducting *TCOs* can be used in developing magnetic sensors and non-volatile memories in spintronics devices with a low energy consumption^7,50^. If *BMPs* are coalescing, even at the room temperature strongest effect of *BMPs* on the transport, magnetization and magnetooptical properties^51^ of magnetic semiconductors can be expected.

## Methods

First alpha silicon nitride (*α-Si*_3_*N*_4_) thin films with a nominal thickness of about 88 nm were deposited in a *Roth* and *Rau AK1000* microwave *PECVD* reaction chamber. Afterwards *ZnO*, *ZnCoO*, and *ZnMnO* thin films with the nominal concentration of 2 at.% and 5 at.% *Co* and *Mn* have been grown on top of *Si*_3_*N*_4_*/p-Si MIS* structures by *PLD* with 700 1 Hz *KrF* excimer laser pulses with energy density of 1.60 Jcm^−2^ to ablate *ZnO*, *ZnMnO*, and *ZnCoO* ceramic targets at a substrate temperature of 550 °C with a constant oxygen flux of 4.50 sccm. Two different oxygen partial pressures, 6.50 × 10^−3^ mbar and 3.91 × 10^−2^ mbar, have been applied to control the concentration of oxygen vacancies in the magnetic *ZnO* thin films. The bottom of the *p-Si* has been coated with gold (*Au*) using *dc* magnetron sputtering at room temperature to form a bottom contact to the *MIS* structure. Circular *dc* magnetron sputtered aluminium dots of different size have been prepared on the *ZnO* films to form the top contacts on the *MIS* structure. For impedance measurements we have chosen *Al* contacts with and area of 5.026 × 10^−7^ m^2^ (*A1*) and of 2.827 × 10^−7^ m^2^ (*A*2).

Structural properties of investigated ten different metal/*n-ZnO* semiconductor/*Si*_3_*N*_4_*Si*_3_*N*_4_ insulator/p-Si semiconductor (*MSIS*) structures, mainly thickness of the *n-ZnO* and *Si*_3_*N*_4_, have been determined using secondary electron microscopy (*SEM*) cross section measurements (Sect. S1). Impedance of the *MSIS* structures with ten different *ZnO*, *ZnCoO*, and *ZnMnO* thin films grown on *Si*_3_*N*_4_*/p-Si* was measured versus voltage (*V*) and versus frequency (*F*) using the *Agilent 4294A* precision impedance analyzer. We determined the bias range for the different regimes in the *MSIS* structure (accumulation, depletion, inversion, strong inversion) by voltage dependent impedance measurements (Sect. S2). Nonlinear behaviour of the frequency dependent capacitance (*C-F*) and conductance (*G-F*) of all *MSIS* structure in strong inversion has been modelled with an equivalent circuit model which accounts for all *RC* elements in the interfaces and layers of the *MSIS* structure. The static dielectric constant of *n-ZnO* has been extracted from modelled capacitance (*C*_*ZnO*_) of completely depleted *n-ZnO* layer of the *MSIS* structure (Sect. S3).

## Supplementary information

Supplementary Information.
