# Supplementary material for: Increased static dielectric constant in ZnMnO and ZnCoO thin films with bound magnetic polarons
Source: Sci Rep. 2020 Apr 21;10:6698. doi: 10.1038/s41598-020-63195-1 (PMC7174340; doi:10.1038/s41598-020-63195-1)
Supplement: Supplementary file 1 — Supplementary Information. [file 41598_2020_63195_MOESM1_ESM.pdf]

Supplementary material: Increased static dielectric constant in *ZnMnO* and *ZnCoO* thin films with bound magnetic polarons

Sahitya V. Vegesna[1,2,*], Vinayak J. Bhat[1,2], Danilo Bürger[3], Jan Dellith[1], Ilona Skorupa[4], Oliver G. Schmidt[5,6], and Heidemarie Schmidt[1,2,3,*]

[1]Leibniz Institute of Photonic Technology, 07745 Jena, Germany
[2]Institute for Solid State Physics, Friedrich Schiller University Jena, 07743 Jena, Germany
[3]Fraunhofer Institute for Electronic Nano Systems, Department Back-End of Line, 09126 Chemnitz, Germany
[4]Institute of Ion Beam Physics and Materials Research, Helmholtz-Research Center Dresden-Rossendorf, 01314 Dresden, Germany
[5]Institute for Integrative Nanosciences, Leibniz Institute for Solid State and Materials Research Dresden, 01069 Dresden, Germany
[6]Material Systems for Nanoelectronics, Technische Universität Chemnitz, 09126 Chemnitz, Germany
Corresponding authors:
[*]SahityaVarma.Vegesna@leibniz-ipht.de
[*]Heidemarie.Schmidt@leibniz-ipht.de

*Note:

We refer to the tables and figures in main manuscript by Table X* and Figure Y*, respectively.

S1 *ZnO* coated Si_3N_4 insulator *MSIS* structure

The nominal thickness of *ZnO* from *PLD* deposition is 120 nm. Scanning electron microscopy (*SEM*) measurement has been carried out with *FET Helois Nanolab G3 UC* system to determine the exact *ZnO* thickness. The excited electron energy is kept at 5 keV and probe current of 13 pA. The secondary electrons are detected with a through the lens detector *TLD*. In order to prevent formation of metallic nanoparticles in magnetic *ZnO*, we kept the substrate temperature around 550°C[2]. Udayakumar et al.[3] detected *ZnCoO* nanoparticles with a size resolution of 500 nm in magnetic *ZnO* by cross-section *SEM*. Using *SEM* focused ion beam (*FIB*) prepared cross section microscopy (Figure S2) we could not detect formation of *Mn* and *Co* nanoparticles larger than 50 nm in magnetic *ZnMnO* and *ZnCoO*, respectively, on Si_3N_4/Si. We can clearly distinguish *Pt* nanoparticles contrast from *Pt* protection layer in Figure S2 used for *SEM FIB* preparation and only columnar *SEM* contrast of crystallite structures

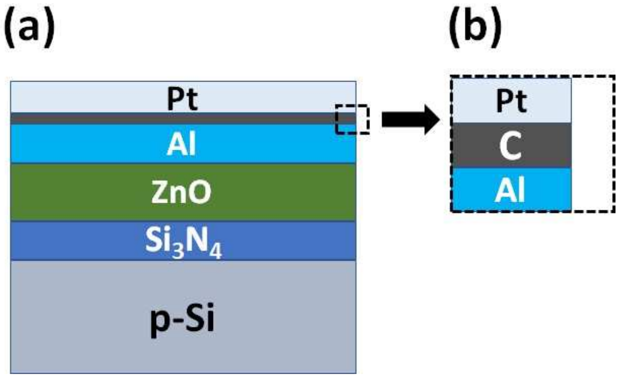

Figure S1. (**a**) *ZnO* and magnetic *ZnO* on $Si_3N_4/p\text{-}Si$ metal insulator semiconductor structure was protected with platinum (*Pt*) layer for secondary electron microscopy (*SEM*) measurements. (**b**) Additionally, a carbon (*C*) layer is coated between platinum and aluminium (*Al*).

Table S1. Measured *ZnO* and Si_3N_4 thickness from *SEM* and calculated Si_3N_4 capacitance value from Si_3N_4 thickness. Relative permittivity of Si_3N_4 is taken as 7.5[1].

Sample	ZnO thickness (nm)	Si_3N_4 thickness (nm)	Si_3N_4 capacitance (mF/m^2)
**ZnO_LP**	93.0	90.9	0.730
**ZnO_HP**	103.4	89.7	0.740
**$Zn_{0.95}Co_{0.05}O$_LP**	120.6	87.7	0.757
**$Zn_{0.95}Co_{0.05}O$_HP**	118.3	81.4	0.815
**$Zn_{0.98}Co_{0.02}O$_LP**	120.3	87.4	0.760
**$Zn_{0.98}Co_{0.02}O$_HP**	118.3	88.1	0.754
**$Zn_{0.95}Mn_{0.05}O$_LP**	116.6	87.3	0.760
**$Zn_{0.95}Mn_{0.05}O$_HP**	117.3	87.3	0.761
**$Zn_{0.98}Mn_{0.02}O$_LP**	120.0	88.0	0.754
**$Zn_{0.98}Mn_{0.02}O$_HP**	101.5	85.6	0.776

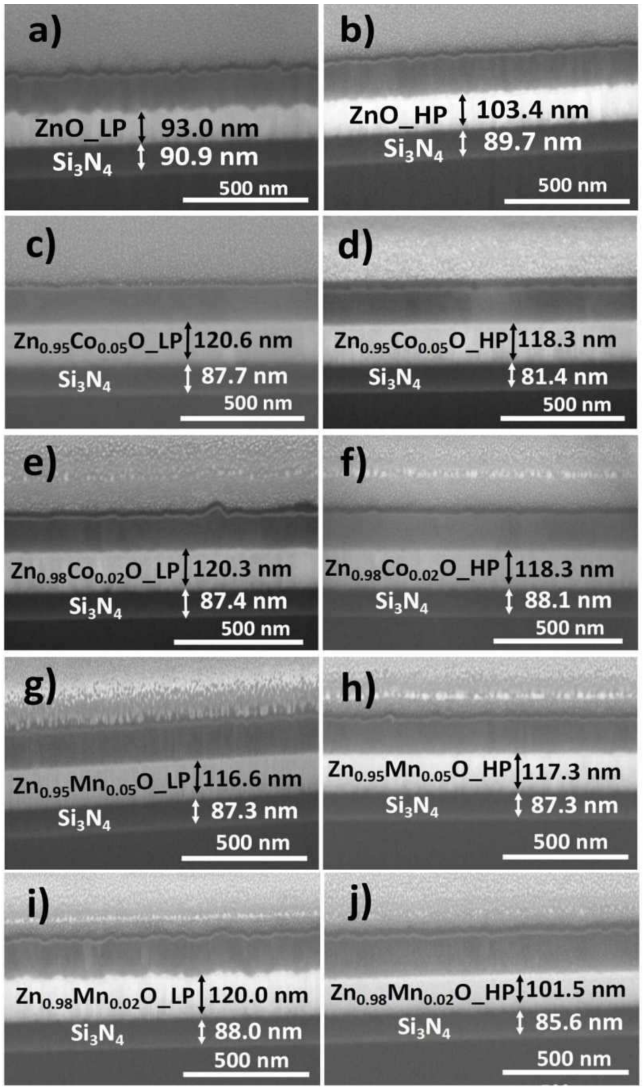

Figure S2. Cross sectional *SEM* images for *ZnO* (**a, b**), *ZnCoO* (**c, d, e, f**), and *ZnMnO* (**g, h, i, j**). Corresponding thickness of *ZnO* and Si_3N_4 is represented on the right side of the arrows.

of *ZnO* and magnetic *ZnO* with preferentially *c*-axis textured growth can be confirmed from our earlier work of *ZnO* thin films on sapphire substrate[4]. ImageJ open software has been used to determine the average thickness of the *ZnO* and Si_3N_4. The obtained thickness is listed in Table S1 and well agrees with the nominal thickness. With the known thickness and the static dielectric constant of Si_3N_4, the calculated Si_3N_4 capacitance (Table S1) and all other parameters of the *MSIS* equivalent circuit model have been used to model the capacitance of the *ZnO* layer and to extract the static dielectric constant of *ZnO* and magnetic *ZnO* thin films.

S2 Voltage dependent impedance

First we discuss the voltage dependent capacitance (*C-V*) and conductance (*G-V*) measured on MSIS structures with ZnO thin films at a constant test frequency (10 kHz) under dark conditions at room temperature. *C-V* and *G-V* are shown in Figure S3 for two different top area contacts namely A1 = 5.026×10^{-7} m^2 (*A1*) and 2.827×10^{-7} m^2 (A2). The area of the unstructured rectangular bottom contact is 0.5 cm x 0.5 cm (250×10^{-7} m^2). The area of the largest top contact is smaller than 2 % of the area of the bottom contact. You *et al.*[5] reported that under application of a bias, electric field is also present in the boundary region outside the area coated by the top electrode. Similarly, the increase in observed capacitance per unit area for larger top contact *A1* (red line) in comparison to the capacitance per unit area for smaller top contact *A2* (blue line) (Figure S3) can be related with the extension of electric field proportional to contact area.

The flat band voltage of the accumulation branch is shifted into the negative bias range due to positively charged defects in Si_3N_4. The flat band voltage position depends on the ramping direction (Figure S3) and range. Here it lies between -2 V and -5 V (Figure 1*). For an applied bias of -15 V mobile positive charges in Si_3N_4 can be accumulated in Si_3N_4 at the ZnO/Si_3N_4 interface and corresponding flat band voltage of the depletion-inversion branch lies in the range from -15 V to -10 V. For an applied bias of +5 V mobile positive charges can be accumulated in the Si_3N_4 at the Si_3N_4/*p-Si* interface.

Here we chose the *MSIS* structure with *ZnO_LP* (Figure S4) to describe the effect of defective Si_3N_4 on the *C-F* and *G-F* behaviour of the *MSIS* structure for accumulation (-20 V), depletion (-5 V), and inversion(+5 V). In accumulation, we measured the frequency dependent capacitance at a large dc bias of -20 V and observed an increase of *ac* capacitance below 100 kHz which is factor 2 larger in accumulation in comparison to capacitance in inversion. Because the constant capacitance at small frequency is usually related with capacitance of the dielectric layer (Si_3N_4), no difference between constant capacitance in accumulation and inversion is expected. One rather expects a constant frequency dependent *ac* capacitance limited which is determined by the thickness and static dielectric constant of the Si_3N_4. However, an increase in *ac* capacitance below 100 kHz in accumulation can be related with the reduction in effective thickness of Si_3N_4 due to mobile defects which are active below 100 kHz and are accumulated in Si_3N_4 at the *n-ZnO*/Si_3N_4 interface and effectively reduce the thickness of insulating Si_3N_4. The frequency dependent capacitance and conductance for accumulation, depletion, and inversion are shown in Figure S4. The conduction in accumulation regime (red scattered line) shows a non-zero offset in the low frequency range. The conduction in inversion (green scattered line) and in depletion (black scattered line) in low frequency range is nearly zero. We relate larger DC conductance below 1 kHz in accumulation with high probability for electron tunnelling from large band gap *n-ZnO* semiconductor into small band gap *p-Si* semiconductor where the carrier injection ratio is large, i.e. injection of electrons from n-ZnO is more dominant than the injection of the holes from p-Si[6]. In depletion (-5 V), we measured frequency dependent capacitance and observed an increase of capacitance below 10 kHz. We explain the increase of capacitance in accumulation (-15 V) and in depletion (-5 V) with frequency dependent mobility of mobile positive charges in Si_3N_4. The more mobile charges contribute to the conductivity in Si_3N_4, the smaller is the effective thickness of insulating Si_3N_4. This influences the capacitance of Si_3N_4. The expected increase in capacitance is shown in Figure S4. Concentration of mobile charges at Si_3N_4/p-Si interface for higher electric field in strong inversion regime is larger (Figure 1*(**c**)) in comparison to depletion regime (Figure 1*(**b**)). Therefore, the possibility of hopping is reduced with increase in positive voltage.

Negligible frequency dependence of Si_3N_4 capacitance on measured capacitance is observed in strong inversion (+5 V). Therefore, the frequency dependent impedance (*C-F*) is measured in strong inversion where conductance per unit area is saturated and where the *ZnO* and magnetic *ZnO* layers in the *MSIS* structures are completely depleted. The corresponding dc voltage is indicated by scattered lines in Figure S3.

In our model, we describe the frequency dependent capacitance (*C-F*) and conductance (*G-F*) in depletion-inversion at strong inversion, i.e. for an applied bias larger than +5 V. *MSIS* structures with *ZnO_LP* (Figure S3(**a**)) and with *ZnO_HP* (Figure S3(**b**)) show a larger reduction of capacitance in strong inversion (+5 V). This is due to the depletion of *ZnO* near ZnO/Si_3N_4 interface during depletion-inversion ramping. *MSIS* structures with magnetic *ZnO* are almost completely depleted before *p-Si* is

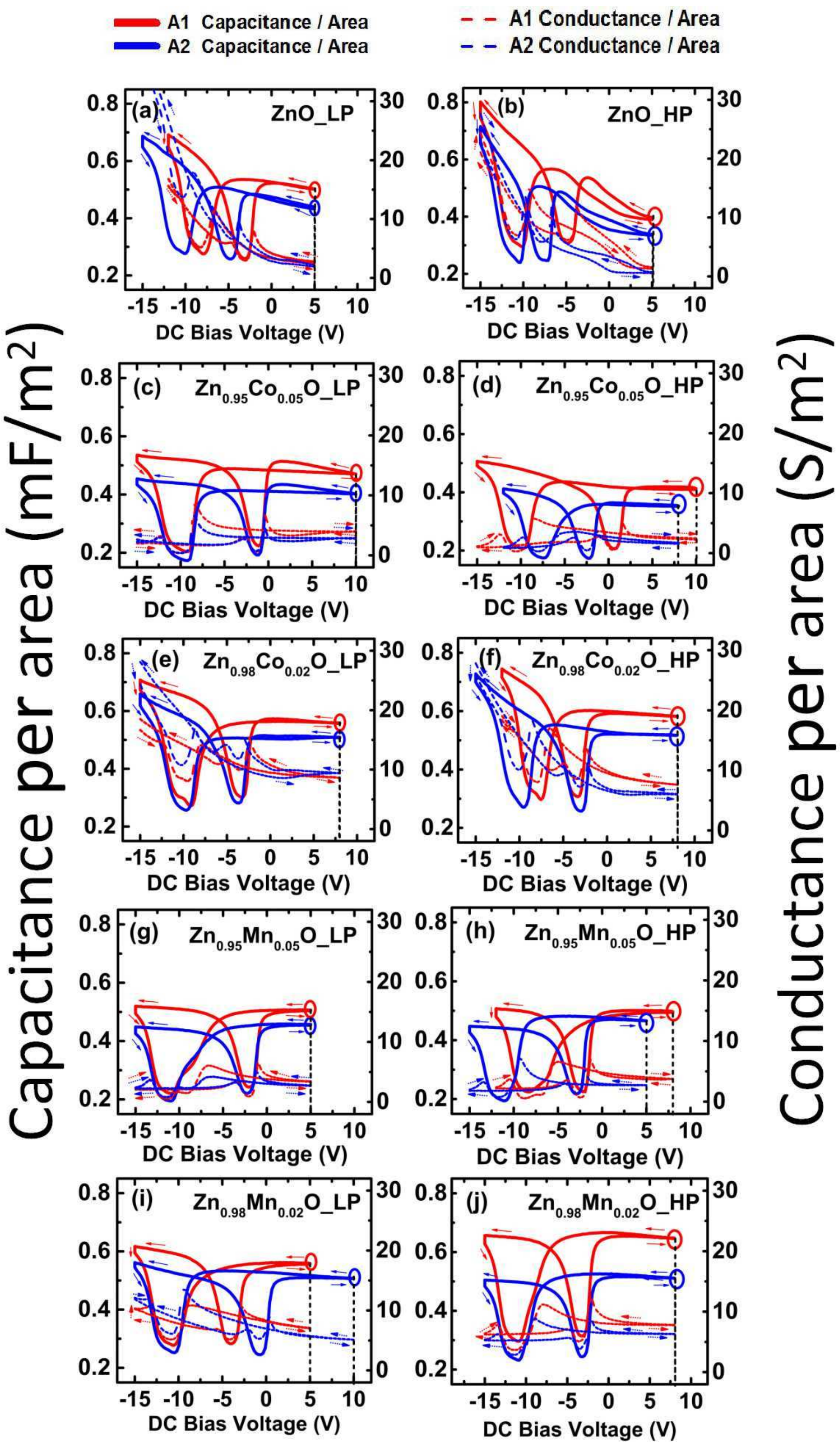

Figure S3. Measured voltage dependent capacitance (*line*) and conductance (*symbol*) for *ZnO* (**a**,**b**), for *ZnCoO* (**c**, **d**, **e**, **f**), and for *ZnMnO* (**g**, **h**, **i**, **j**) with *A1* (5.026E-7 m^2, red lines and symbols) and *A2* (2.827E-7 m^2, blue line and symbols). The voltage has been ramped (arrows from left to right) from accumulation (Figure 1**a**) to depletion (Figure 1**b**) to inversion (Figure 1**c**) and (arrows from right to left) back from inversion to depletion and accumulation. *C-V* measurements were taken at a test frequency of 10 kHz only up to a voltage where the inversion has been reached. This is indicated by scattered lines. For both ramping directions the minimum of the flatband voltage lies in the negative bias range. *LP* samples with 6.50E-3 mbar and *HP* samples with 3.91E-2 mbar are shown in left and right of figure columns, respectively.

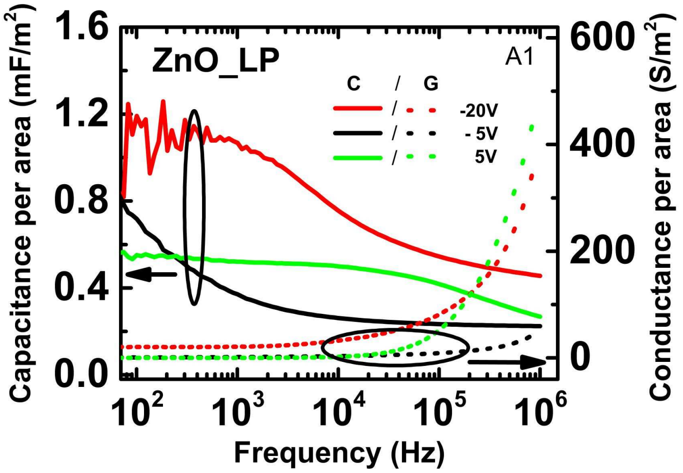

Figure S4. Measured frequency dependent capacitance (*C-F*) and conductance (*G-F*) for *MSIS* structure with *ZnO_LP* sample in accumulation (-20 V), depletion (-5 V), and inversion (+5 V).

Table S2. Inversion regime circuit impedance parameters of individual layer of reference sample *Al/Si*$_3$*N*$_4$*/p-Si/Au* for *Al/Si*$_3$*N*$_4$ interface, *Si*$_3$*N*$_4$, *p-Si* and *p-Si/Au* interface.

Parameter	Description
$\mathbf{C}_{Al}$, $\mathbf{R}_{Al}$	Interface capacitance and resistance between aluminium (Al) top contact and insulator
$\mathbf{C}_i$	Insulator capacitance (Non leaky Si_3N_4 insulator)
$\mathbf{C}_{dep}$	Depletion layer capacitance in semiconductor.
$\mathbf{C}_{ss}$, $\mathbf{R}_{ss}$	Surface state capacitance and resistance near the insulator and p-Si interface.
$\mathbf{C}_{fss}$, $\mathbf{R}_{fss}$	Fast Surface state capacitance and resistance at the insulator and p-Si interface.
$\mathbf{R}_s$	Combination of p-Si Semiconductor resistance and contact resistances.
$\mathbf{C}_{st}$, $\mathbf{R}_{st}$	Interface Schottky capacitance and Schottky resistance between p-Si and gold (Au) bottom contact.

in strong inversion.

S3 Small signal impedance analysis in strong inversion

In the following the small signal impedance of *MIS* and of *MSIS* structures is analyzed in strong inversion. The modelled parameters of the *MIS* structure (reference samples) have been used afterwards as an estimate for the corresponding parameters of the *MSIS* structures. The modelling of small signal impedance of the *MSIS* structure always starts in the high frequency range where the leaky Si_3N_4 does not dominate frequency dependent small signal impedance. Afterwards the small signal impedance has been modelled in the whole frequency range. C_{ZnO} is the parameter which is used to extract the static dielectric constant of the *ZnO* layer in the *MSIS* structures

S3.1 Reference MIS structure in the whole frequency range

The algorithm for flow chart (Figure S7) contains 5 steps

- Step 1: Values of insulator capacitance C_i and depletion capacitance C_{dep} parameters are taken from the calculated value. These two parameters are not varied throughout the modelling.

- Step 2: Instead of considering all model parameters simultaneously, we advanced in our modelling by adding one block in every step to the circuit in course of leading up to the result. First, we modelled by considering insulator capacitance C_i in series with depletion capacitance C_{dep} and surface state capacitance C_{ss} and surface state resistance R_{ss} along with semiconductor resistor R_s. Variable parameters are changed till a decent fit is obtained.

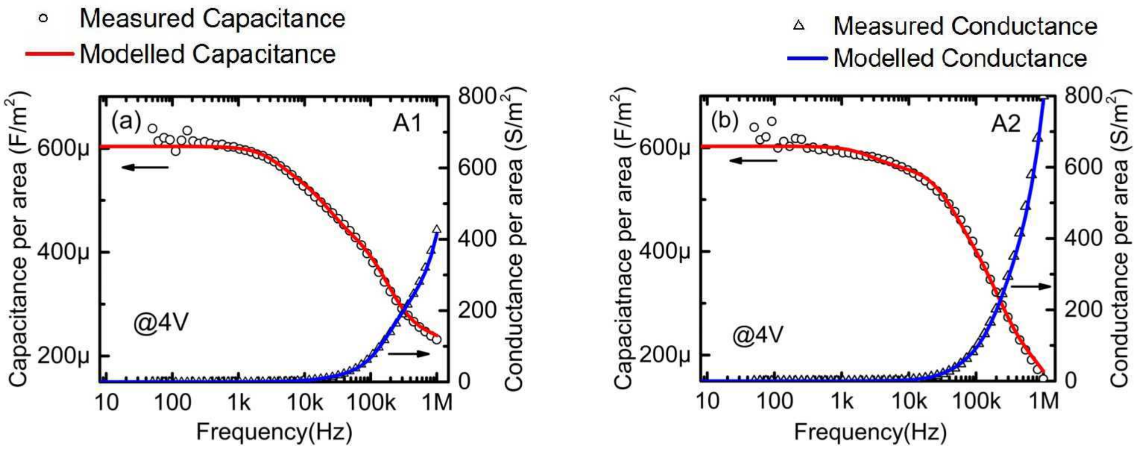

Figure S5. Experimental and modelled frequency dependent capacitance and conductance for the inversion regime of reference sample top contact area sizes (**a**) *A1* (5.026×10^{-7} m^2) and (**b**) *A2* (2.827×10^{-7} m^2).

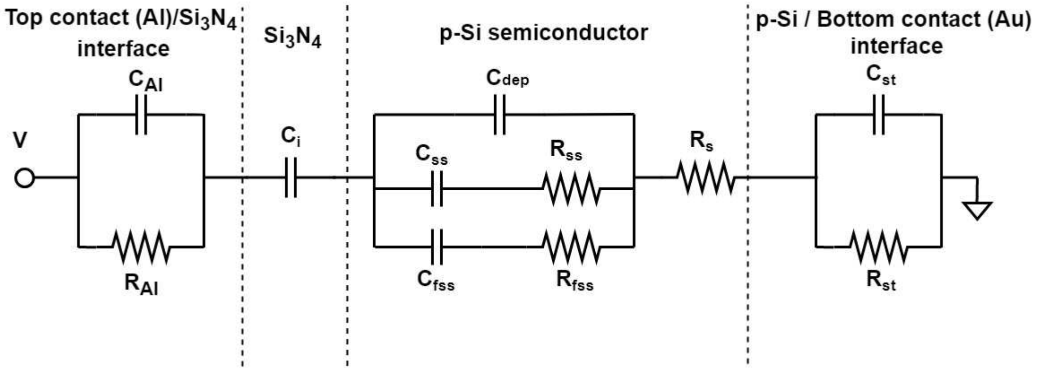

Figure S6. Equivalent circuit model for reference sample *Al/Si*$_3$*N*$_4$*/p-Si* in inversion with *RC* elements for *Al/Si*$_3$*N*$_4$ interface, *Si*$_3$*N*$_4$, *p-Si* and *p-Si/Au* interface regime from left to right.

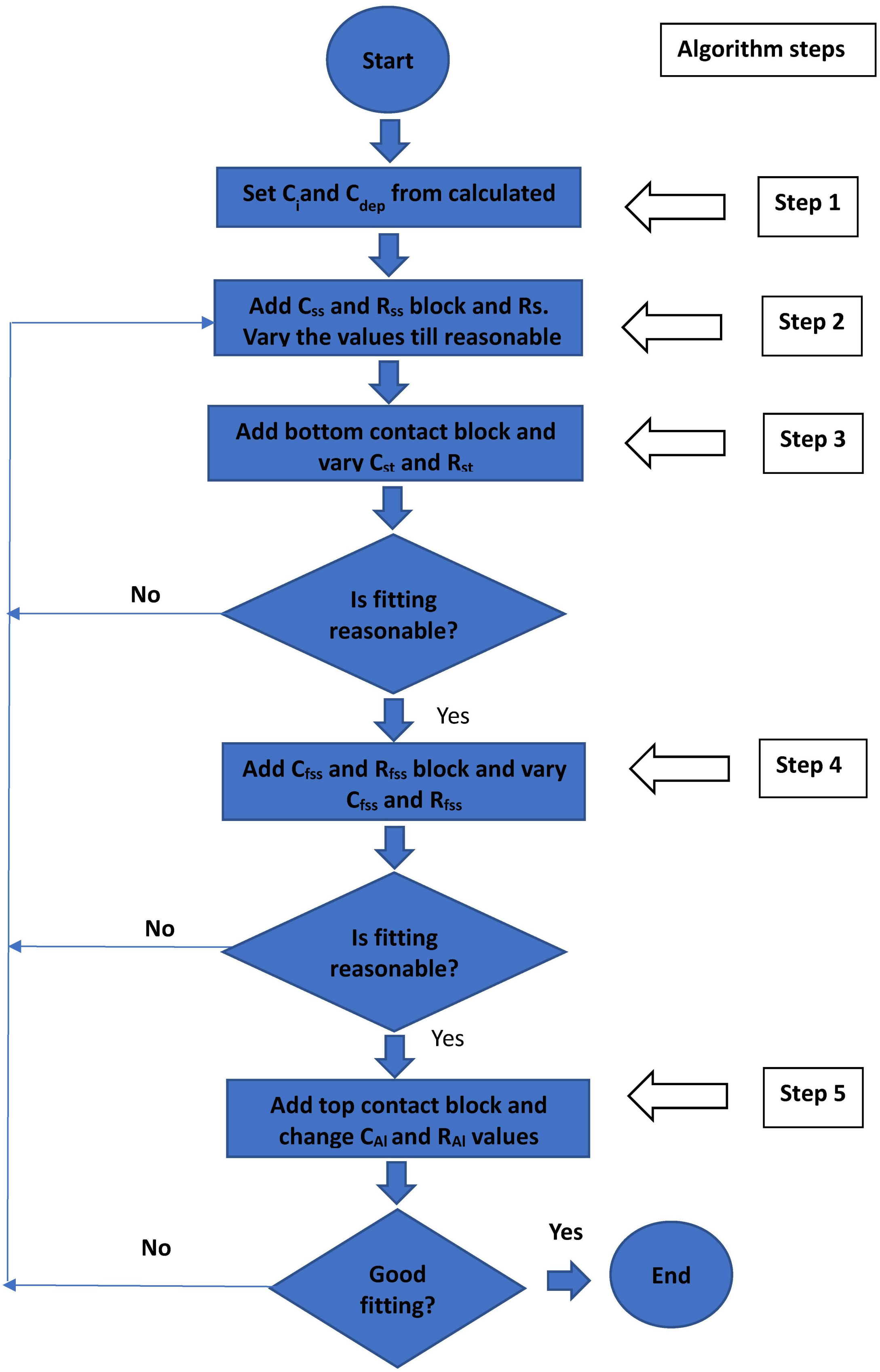

Figure S7. Flow chart for the for frequency dependent impedance modelling of reference sample *Al/*Si_3N_4*/p-Si*.

Table S3. The description of impedance elements for equivalent circuit in Figure S9 of *Al/ZnO/Si_3N_4/p-Si/Au* (M*SIS*) in inversion regime for *Al/ZnO* interface, *ZnO*, *Si_3N_4*, *p-Si* and *p-Si/Au* interface.

**Parameter**	Description
$\mathbf{C}_{Al}$, $\mathbf{R}_{Al}$	Interface capacitance and resistance between aluminium (Al) top contact and insulator
$\mathbf{C}_{ZnO}$, $\mathbf{R}_{ZnO}$	ZnO depletion capacitance and resistance.
$\mathbf{C}_{Zss}$, $\mathbf{R}_{Zss}$	Surface state charges impedance at the interface of ZnO and Si_3N_4 insulator.
$\mathbf{C}_i$	Insulator capacitance (Non leaky Si_3N_4 insulator)
$\mathbf{C}_{dep}$	Depletion layer capacitance in semiconductor.
$\mathbf{C}_{ss}$, $\mathbf{R}_{ss}$	Surface state capacitance and resistance near the insulator and p-Si interface.
$\mathbf{C}_{fss}$, $\mathbf{R}_{fss}$	Fast Surface state capacitance and resistance at the insulator and p-Si interface.
**Rs**	Combination of p-Si Semiconductor resistance and contact resistances.
$\mathbf{C}_{st}$, $\mathbf{R}_{st}$	Interface Schottky capacitance and Schottky resistance between p-Si and gold (Au) bottom contact.

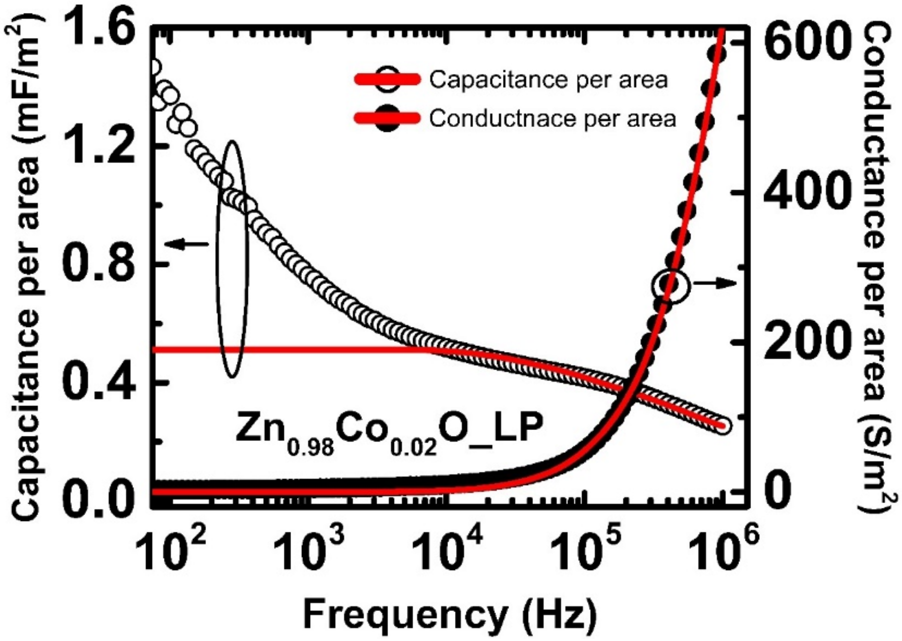

Figure S8. One example to see the behaviour of high frequency range frequency dependent capacitance (*C-F*) and conductance (*G-F*) of sample $Zn_{0.98}Co_{0.02}O$_LP with non-leaky *Si_3N_4*.

- Step 3: In this step we add the bottom Schottky contact capacitance C_{st} and Schottky resistance R_{st}. Fitting is performed by varying C_{st} and R_{st} along with the other variable parameters till a reasonable fit for the *C-F* characteristic curve is obtained.

- Step 4: Adding fast surface state capacitance C_{fss} and resistance R_{fss} to the circuit and we perform the same procedure as that of previous steps.

- Step 5: In the final step, aluminium top contact, *Si_3N_4* interface layer capacitance resistance block C_{Al} and R_{Al} is introduced in the circuit model for C-F curve fitting. We obtained a good fitting result with reasonable values for the parameters.

S3.2 ZnO coated MSIS structure in high frequency range (>10 kHz)

The algorithm for flow chart (Figure S10) contains 5 steps.

- Step 1: Calculated values of *Si_3N_4* capacitance C_i from the obtained thickness from *SEM* measurement in Sec. **S1** and the values of reference sample model parameters are taken. The *Si_3N_4* capacitance value C_i , *p-Si* depletion capacitance

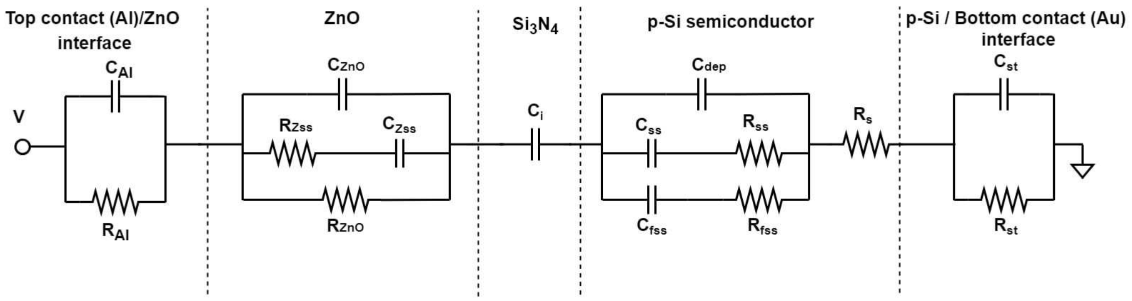

Figure S9. Equivalent circuit model for *Al/ZnO/*Si_3N_4*/p-Si/Au* Metal insulator semiconductor in inversion regime with *RC* elements for *Al/ZnO* interface, *ZnO*, Si_3N_4, *p-Si* and *p-Si/Au* interface from left to right. Note that with this equivalent circuit model Si_3N_4 can not be considered as a leaky dielectric.

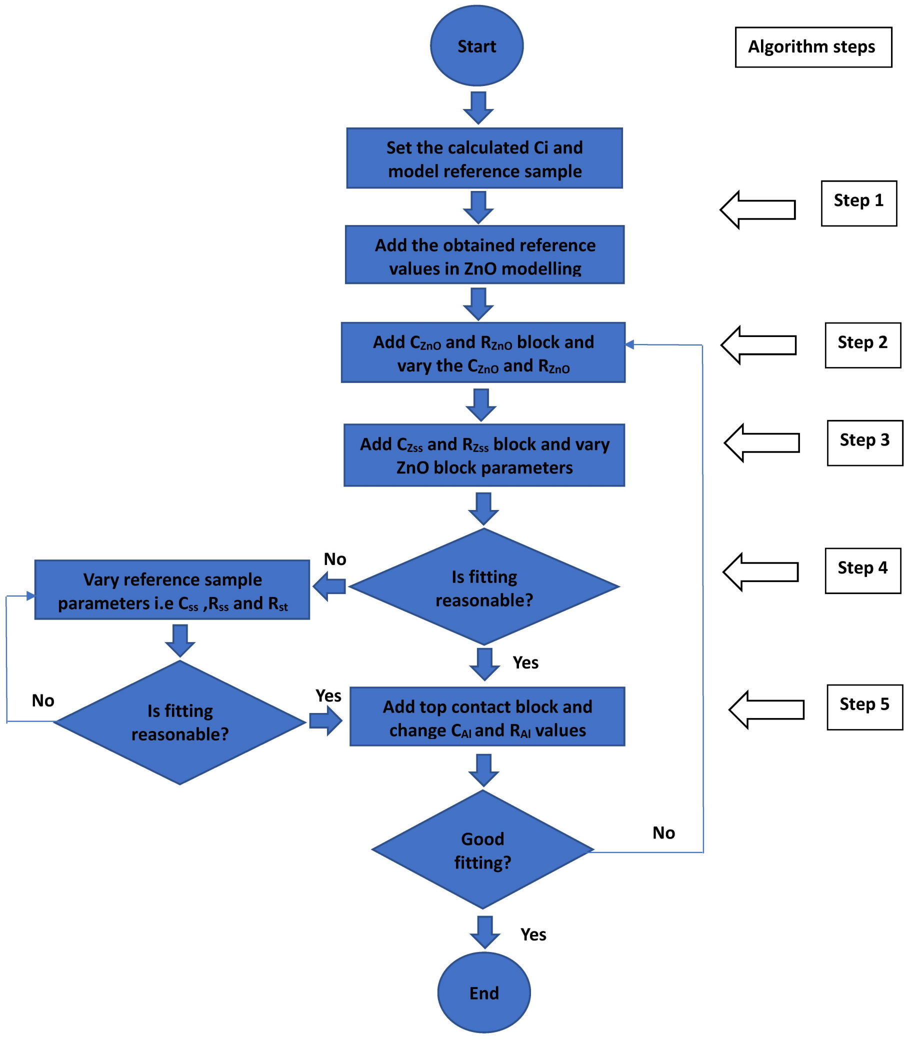

Figure S10. Flow chart for the for modelling frequency dependent impedance of *MSIS* structure with a non-leaky Si_3N_4.

Table S4. The description of impedance elements for equivalent circuit in Figure S12 of *Al/ZnO/*Si_3N_4*/p-Si/Au* (M*SIS*) in inversion regime for *Al/ZnO* interface, *ZnO*, *ZnO/*Si_3N_4 interface, Si_3N_4, Si_3N_4*/p-Si* interface, *p-Si* and *p-Si/Au* interface

Parameter	Description
C_{Al}, R_{Al}	Interface capacitance and resistance between aluminium (Al) top contact and insulator
C_{ZnO}, R_{ZnO}	ZnO depletion capacitance and resistance.
C_{Zss}, R_{Zss}	Surface state charges impedance at the interface of ZnO and Si_3N_4 insulator.
R_{ZI}	Part of a broken ac channel with same dc conduction in leaky Si_3N_4 insulator at the interface of ZnO and Si_3N_4. (R_{ZI} = R_{IS}).
C_i	Insulator capacitance (Non leaky Si_3N_4 insulator)
R_{SI}	Part of a broken ac channel with same dc conduction in leaky Si_3N_4 insulator at the interface of Si_3N_4 and p-Si. (R_{ZI} = R_{IS}).
C_{dep}	Depletion layer capacitance in semiconductor.
C_{ss}, R_{ss}	Surface state capacitance and resistance near the insulator and p-Si interface.
C_{fss}, R_{fss}	Fast Surface state capacitance and resistance at the insulator and p-Si interface.
**Rs**	Combination of p-Si Semiconductor resistance and contact resistances.
C_{st}, R_{st}	Interface Schottky capacitance and Schottky resistance between p-Si and gold (Au) bottom contact.

value C_{dep} and the Schottky capacitance at the interface of the p-Si and bottom contact capacitance C_{st} is not varied throughout the modelling procedure.

- Step 2: Instead of considering all the ZnO parameter simultaneously in the modelling, we add the C_{ZnO} and R_{ZnO} parallel block in this step. C_{ZnO} and R_{ZnO} parameters are varied till a decent fit is obtained.

- Step 3: In this step the surface capacitance C_{Zss} and resistance R_{Zss} is added to the circuit model. Fitting is performed by varying all *ZnO* parameters till a reasonable fit is obtained.

- Step 4: If reasonable fit is not obtained, the variable parameters from the reference sample C_{ss}, R_{ss} and R_{st} is varied till a good fit is obtained.

- Step 5: Aluminium (*Al*) top contact and *ZnO* interface capacitance C_{Al} and resistance R_{Al} is introduced to the circuit model. Same procedure as the previous steps is performed till a good fitting result with reasonable parameter values are obtained.

S3.3 ZnO coated MSIS structure in the whole frequency range Parameters obtained, Table S2 and S3

The algorithm for flow chart (Figure S13) contains 5 steps.

- Step 1: The parameters (Table S3) modelled from high frequency range (>10 kHz) (Sect **S3.2**) are kept same for the parameters in Table S4

- Step 2: Frequency dependent leaky insulator capacitance has been introduced into the modelling replacing static insulator capacitor in Section **S3.2** using Eq. S1

- Step 3: A broken *ac* channel with same *dc* conduction and for small signal equivalent circuit has to be considered for leaky Si_3N_4. The *ac* channel resistances are added at the interfaces of *ZnO/*Si_3N_4 (R_{ZI}) and Si_3N_4*/p-Si* (R_{IS}) keeping same values for R_{ZI} and R_{IS} as they belong to same *dc* conduction.

- Step 4: In this step, it was necessary to add the fast surface capacitance C_{Zfss} and resistance R_{Zfss} to the circuit model replacing R_{ZnO} (Figure S9). Fitting is performed by slightly varying all ZnO parameters till a reasonable fit is obtained.

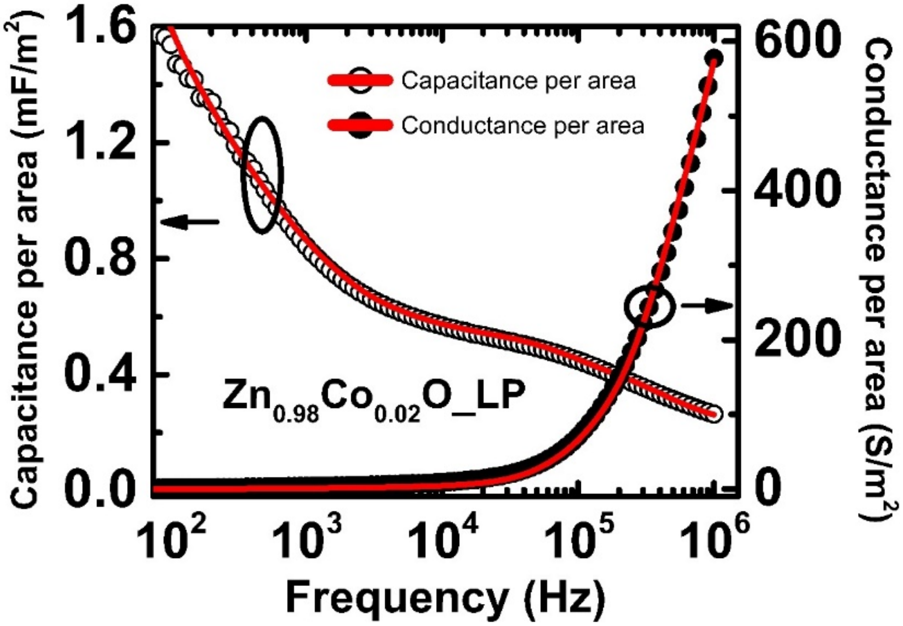

Figure S11. One example to see the behaviour of whole frequency range frequency dependent capacitance (C-F) and conductance (G-F) of sample $Zn_{0.98}Co_{0.02}O$_LP with leaky Si_3N_4.

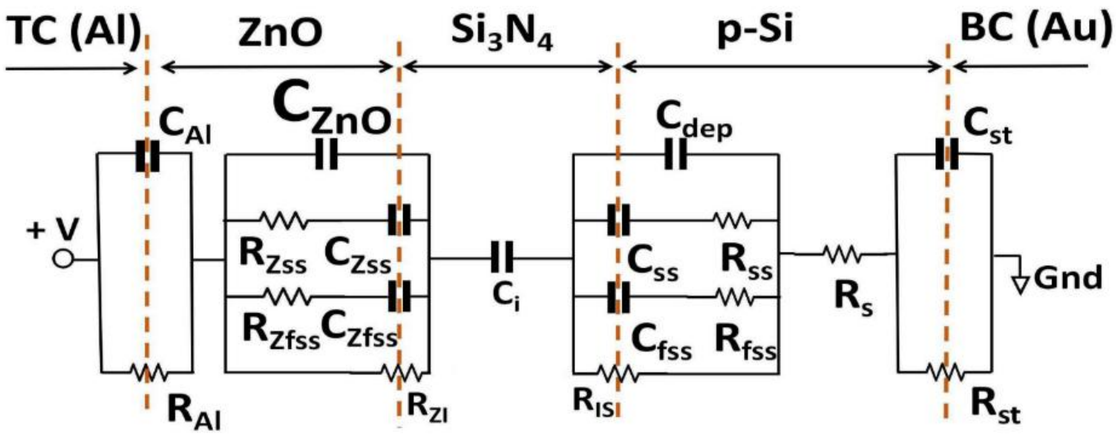

Figure S12. Equivalent circuit model for *Al/ZnO/Si_3N_4/p-Si/Au* Metal insulator semiconductor in inversion regime with *RC* elements for *Al/ZnO* interface, *ZnO*, *Si_3N_4*, *p-Si* and *p-Si/Au* interface from left to right. Note that with this equivalent circuit model *Si_3N_4* can be considered as a leaky dielectric which reveals *ac* conductivity at the *ZnO/Si_3N_4* (R_{ZI}) and at the *Si_3N_4/p-Si* (R_{IS}) interface.

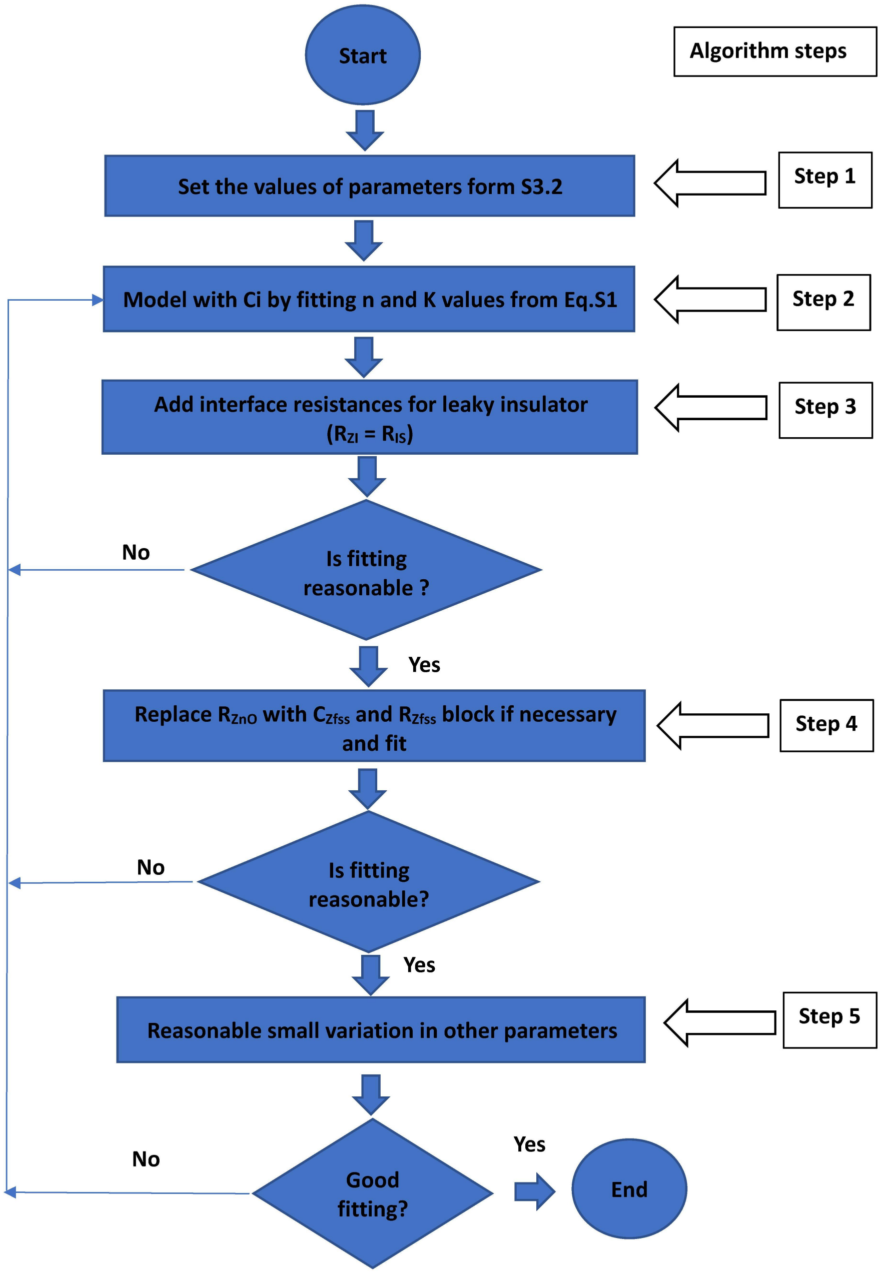

Figure S13. Flowchart describing steps to follow for impedance modelling in whole range after obtaining values from high frequency impedance modelling.

Table S5. Modelled (*RC*) elements of *MSIS* equivalent circuit model in inversion (+5 V) of *MSIS* structure with *ZnO*, *ZnCoO* (5 at. %), *ZnCoO* (2 at. %), *ZnMnO* (5 at. %), and *ZnMnO* (2 at. %) with top contact area *A2* (2.827E-7 m^2) and *A1* (5.026E-7 m^2). The value of C_{dep} is 0.37 mF/m^2, C_{fss} is 0.09 mF/m^2, R_{fss} is 0.20 mohm-m^2, C_{st} is 2.40 mF/m^2 and R_{zfss} is 0.01 mohm-m^2. The unit of C_{Al}, C_{Zss}, C_{Zfss}, and C_{ss} is mF/m^2 and the unit of R_{Al}, R_{Zss}, R_{ZI}, R_{IS}, R_{ss}, R_s, and R_{st} is mohm-m^2.

Sample	Contact	C_{Al}	R_{Al}	C_{Zss}	R_{Zss}	C_{Zfss}	$R_{ZI} = R_{IS}$	C_{ss}	R_{ss}	R_s	R_{st}
**ZnO_LP**	**A1**	8.00	2.00	1.65	0.60	1.00	7000	3.15	0.34	0.005	0.70
	**A2**	6.80	3.02	0.60	0.75	0.20	2500	2.90	0.28	0.005	0.54
**ZnO_HP**	**A1**	4.50	1.00	0.29	1.50	0.08	300000	2.90	0.27	0.005	0.36
	**A2**	9.00	0.80	0.09	2.00	0.02	200000	2.20	0.26	0.02	0.31
**$Zn_{0.95}Co_{0.05}O$_LP**	**A1**	6.20	6.00	3.70	1.20	0.08	30000	2.40	0.49	0.03	2.10
	**A2**	4.00	4.20	0.70	2.80	0.08	30000	2.20	0.46	0.04	1.10
**$Zn_{0.95}Co_{0.05}O$_HP**	**A1**	8.00	8.00	0.56	2.80	0.07	40000	1.80	0.68	0.05	1.40
	**A2**	4.90	7.00	0.03	0.61	0.01	40000	1.75	0.64	0.07	0.82
**$Zn_{0.98}Co_{0.02}O$_LP**	**A1**	7.00	2.50	2.50	0.60	4.90	25	2.40	0.50	0.03	0.70
	**A2**	10.00	1.50	0.90	0.80	1.40	32	2.40	0.44	0.06	1.10
**$Zn_{0.98}Co_{0.02}O$_HP**	**A1**	6.00	1.00	10.90	0.25	1.90	15	2.70	0.40	0.02	0.85
	**A2**	6.50	3.50	2.90	0.35	2.00	58	2.30	0.37	0.03	0.82
**$Zn_{0.95}Mn_{0.05}O$_LP**	**A1**	5.50	3.00	3.30	0.40	0.50	9000	2.40	0.40	0.02	0.80
	**A2**	5.50	4.50	0.90	0.80	0.10	4000	2.40	0.36	0.03	0.85
**$Zn_{0.95}Mn_{0.05}O$_HP**	**A1**	5.00	4.00	1.60	0.60	0.30	9000	2.40	0.39	0.01	0.80
	**A2**	6.50	2.00	0.40	0.80	0.60	50000	2.40	0.32	0.01	0.65
**$Zn_{0.98}Mn_{0.02}O$_LP**	**A1**	5.70	1.00	3.40	0.50	4.30	22	2.50	0.40	0.03	1.00
	**A2**	5.80	2.50	0.50	1.40	1.60	36	2.30	0.30	0.03	0.65
**$Zn_{0.98}Mn_{0.02}O$_HP**	**A1**	20.00	0.20	20.00	0.12	2.20	16	2.50	0.42	0.02	0.50
	**A2**	7.00	2.00	0.80	0.65	1.00	32	2.10	0.44	0.05	1.10

- Step 5: If reasonable fit is not obtained, the variable parameters in the modelling have been slightly modified till a good fit is obtained.

S3.4 Detailed analysis of MSIS structures with leaky Si_3N_4

In the following we discuss how the small signal impedance of MSIS structures with mobile positive charges in Si_3N_4 have been modelled in in strong inversion in the small frequency range. Experimental and modelled impedance of *MSIS* structures *ZnCoO_LP* and *ZnCoO_HP* with 5 at. % and 2 at. % *Co* concentration is shown in Figure S14 (**c, d, e** and **f**) and of *MSIS* structures *ZnMnO_LP* and *ZnMnO_HP* for 5 at. % and 2 at. % *Mn* concentration are shown in Figure S14 (**g, h, i** and **j**). Modelled (*RC*) elements are listed in Table S5, namely capacitance in units of Farad per area and resistance in units of Ohm times unit area. The fast surface state impedance C_{fss} is 0.09 mF/m^2, R_{fss} is 0.20 mohm-m^2 and bottom contact Schottky barrier capacitance C_{st} is 2.4 mF/m^2, p-Si depletion capacitance C_{dep} is 0.374 mF/m^2, fast surface state resistance R_{zfss} is 0.01 mohm-m^2 are same for all the ten samples.

The frequency dependent insulator capacitance is given by the following equation[7].

$$C_i(\omega) = C_i(\infty)(1 + K\omega^{-2n}) \tag{S1}$$

where $C_i(\omega)$ is frequency (ω) dependent insulator capacitance at small signal *ac* voltage (0.01 V) and $C_i(\infty)$ is the capacitance of the insulator at large frequencies where the influence of defects on impedance measurements is negligible. Fitting parameters *n* & *K* and electricfield in Si_3N_4 are listed in Table S6. The electricfield across Si_3N_4 has been calculated from the applied voltage and thickness measured from *SEM* for respective samples. Modelled Jonscher response[7] (n) of Si_3N_4 is between 0.20 and 0.32 and agrees with theoretical (n) limit, namely $0 < n < 1$, for all the samples. K is a fitting parameter which is inversely proportional to the space charge effect on the interfaces of ZnO/Si_3N_4 and $Si_3N_4/p\text{-}Si$. Here the interface space charge region resistance is modelled with resistances R_{ZI} and R_{IS}. We see the inverse proportionality between modelled interface resistances and modelled parameter K. The larger increase in capacitance for low frequencies in 2 % *Co* (Figure S14(**e**) and S14(**f**)) and 2 % *Mn* (Figure S14(**i**) and S14(**j**)) have larger K and smaller resistances (R_{ZI} and R_{IS}) in comparison to capacitance for low frequencies in 5 % *Co* (Figure S14(**c**) and S14(**d**)) and 5 % *Mn* (Figure S14(**g**) and S14(**h**)) with smaller K and larger resistances (R_{ZI} and R_{IS}). The resistances R_{ZI} and R_{IS} decrease with increasing leakiness of the Si_3N_4 insulator.

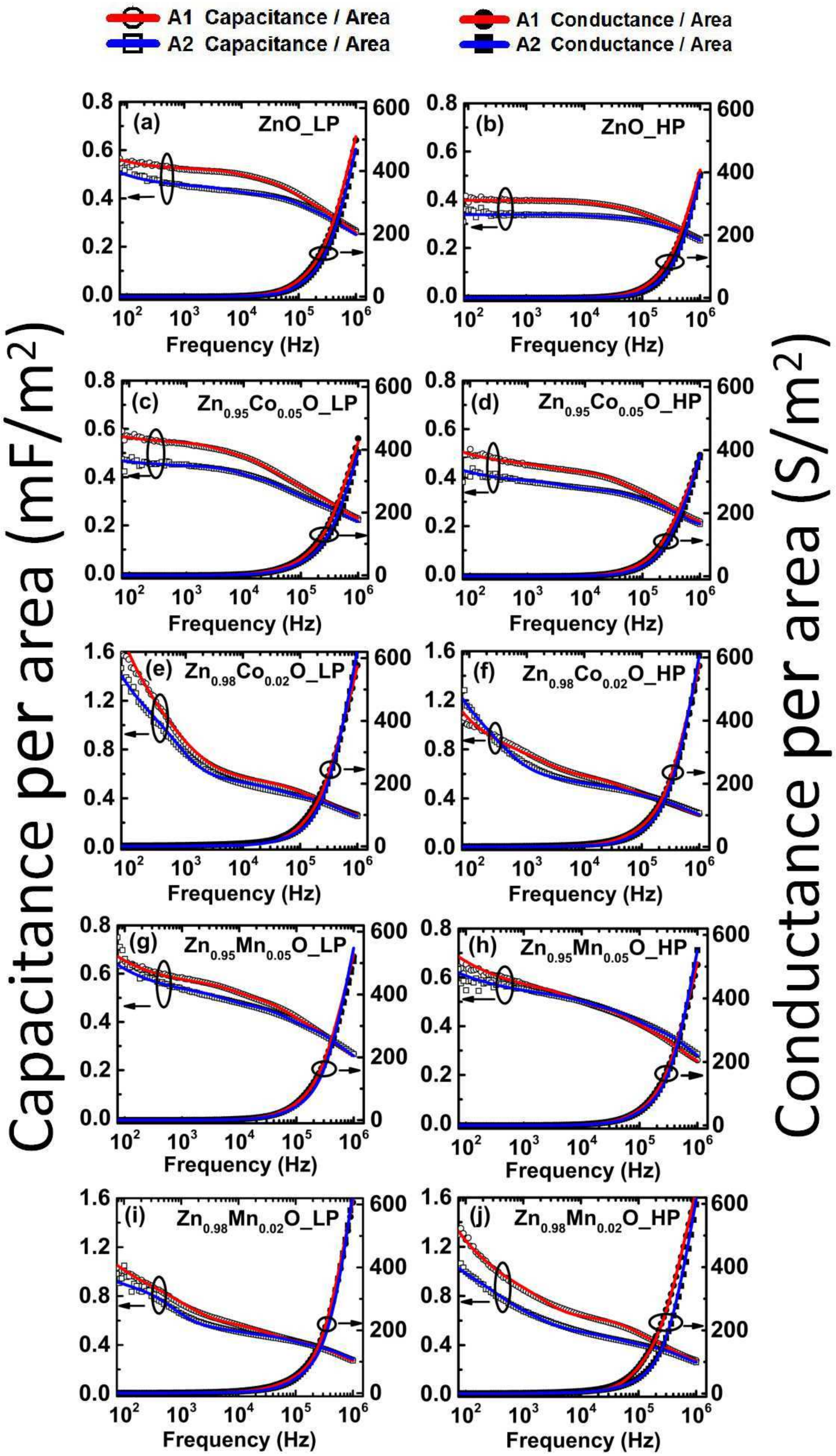

Figure S14. Measured (*symbols*) and modelled (*solid lines*) frequency dependent capacitance (*C-F*) and conductance (*G-F*) of *MSIS* structure with *ZnO* (**a, b**), *ZnCoO* (**c, d, e, f**), and *ZnMnO* (**g, h, i, j**) in strong inversion with A1 (red) and A2 (blue) top contact area. The constant *dc* measurement bias for area *A1* and *A2* is indicated in Figure S3. *MSIS* structures with *ZnO* thin films which have been grown with 6.50E-3 mbar and 3.91E-2 mbar oxygen partial pressure are shown in left and right column, respectively.

Table S6. Model parameters describing increase in capacitance of a defective insulator with decreasing test frequency according to Eq. S1.

Sample	Contact	2n	K	Resistance R_{ZI} =R_{IS} (mohm-m^2)	Electric field (MV/m)
ZnO_LP	A1	0.62	5.50	7000	55.0
	A2	0.61	9.50	2500	55.0
ZnO_HP	A1	0.40	0.01	300000	55.7
	A2	0.40	0.01	200000	55.7
$Zn_{0.95}Co_{0.05}O$_LP	A1	0.60	4.00	30000	113.0
	A2	0.58	4.00	30000	113.0
$Zn_{0.95}Co_{0.05}O$_HP	A1	0.62	13.00	40000	122.0
	A2	0.61	15.00	40000	98.3
$Zn_{0.98}Co_{0.02}O$_LP	A1	0.53	32.00	25	91.6
	A2	0.56	28.00	32	91.6
$Zn_{0.98}Co_{0.02}O$_HP	A1	0.60	18.00	15	90.8
	A2	0.57	22.00	58	90.8
$Zn_{0.95}Mn_{0.05}O$_LP	A1	0.56	12.00	9000	57.3
	A2	0.55	15.00	4000	57.3
$Zn_{0.95}Mn_{0.05}O$_HP	A1	0.55	17.00	9000	91.6
	A2	0.50	10.00	50000	57.3
$Zn_{0.98}Mn_{0.02}O$_LP	A1	0.64	22.00	22	56.8
	A2	0.64	17.00	36	113.0
$Zn_{0.98}Mn_{0.02}O$_HP	A1	0.56	25.00	16	93.5
	A2	0.68	23.00	32	93.5

Table S7. Cut off frequencies in units of kHz and MHz of the (*RC*) elements in the *MSIS* equivalent circuit model (Figure 2*(**b**)) of all ten investigated *MSIS* structures with top contact area size *A1* (5.026E-7 m^2) and *A2* (2.827E-7 m^2).

		Schottky barrier		Semiconductor surface state defects			
				Static defects		Mobile defects	
		TC	BC				
Sample	Contact	Al/ZnO	p-Si/Au	ZnO/Si_3N_4	Si_3N_4/p-Si	ZnO/Si_3N_4	Si_3N_4/p-Si
		$C_{Al} \cdot R_{Al}$ (kHz)	$C_{st} \cdot R_{st}$ (kHz)	$C_{Zss} \cdot R_{Zss}$ (kHz)	$C_{ss} \cdot R_{ss}$ (kHz)	$C_{Zfss} \cdot R_{Zfss}$ (MHz)	$C_{fss} \cdot R_{fss}$ (MHz)
ZnO_LP	A1	11.4	92.1	680.1	148.6	39.6	8.8
	A2	8.1	116.3	837.6	964.5	79.6	8.8
ZnO_HP	A1	32.1	184.2	365.6	203.3	198.9	8.8
	A2	22.1	213.9	884.2	278.2	795.8	8.8
$Zn_{0.95}Co_{0.05}O$_LP	A1	4.3	35.8	35.8	135.3	198.9	8.8
	A2	9.5	60.3	81.2	157.3	198.9	8.8
$Zn_{0.95}Co_{0.05}O$_HP	A1	2.5	47.4	101.5	130.0	4.5	8.8
	A2	4.6	80.9	87.1	142.1	159.1	8.8
$Zn_{0.98}Co_{0.02}O$_LP	A1	9.9	94.7	106.1	132.6	3.2	8.8
	A2	10.6	60.3	221.0	150.0	11.4	8.8
$Zn_{0.98}Co_{0.02}O$_HP	A1	26.5	78.0	58.5	147.4	8.4	8.8
	A2	7.0	80.9	156.8	187.0	7.9	8.8
$Zn_{0.95}Mn_{0.05}O$_LP	A1	9.6	82.9	120.6	165.8	31.8	8.8
	A2	6.4	78.0	221.0	184.2	159.1	8.8
$Zn_{0.95}Mn_{0.05}O$_HP	A1	7.9	82.9	165.8	166.6	53.0	8.8
	A2	12.2	102.0	331.6	203.0	26.5	8.8
$Zn_{0.98}Mn_{0.02}O$_LP	A1	18.6	66.5	93.6	159.1	3.7	8.8
	A2	11.0	102.0	227.4	230.6	9.9	8.8
$Zn_{0.98}Mn_{0.02}O$_HP	A1	79.6	132.6	66.3	151.6	7.2	8.8
	A2	11.3	60.3	306.1	172.2	15.9	8.8

The frequency dependent impedance measurements on the reference sample $Si_3N_4/p\text{-}Si$ (Figure S5) in the strong inversion regime shows a saturated capacitance at low frequencies (100 Hz) equivalent to the non-leaky capacitance of insulating Si_3N_4. Only after the pulsed laser deposition (*PLD*) of *ZnO* on $Si_3N_4/p\text{-}Si$, low frequency capacitance of the structure is increasing (Figure S14) more than the expected saturation capacitance of insulating Si_3N_4. This clearly indicates that the properties of the Si_3N_4 insulator are changed by the deposition of *ZnO* on $Si_3N_4/p\text{-}Si$ during the pulsed laser deposition process at a substrate temperature of 550°C.[8] Typically, one would expect outdiffusion of nitrogen from Si_3N_4 during *PLD*. However, the outdiffusion of nitrogen is suppressed if a larger oxygen partial pressure is applied during the *PLD* process. This is seen in Figure S14 where the increase of low-frequency capacitance due to defect formation in Si_3N_4 is larger in the low pressure (*LP*) samples in comparison to the high pressure (*HP*)-samples, except the $Zn_{0.98}Mn_{0.02}O\_LP$ sample (Figure S14(**i**)) which shows a smaller increase of low-frequency capacitance in comparison to the low-frequency capacitance of the $Zn_{0.98}Mn_{0.02}O\_HP$ sample (Figure S14(**j**)). The composition of Si_3N_4 can also be changed by the diffusion of impurity atoms from the *ZnO* layer into the underlying Si_3N_4. For example, vacancies can be formed in Si_3N_4 if the lattice of Si_3N_4 is locally enlarged at the substrate temperature of 550°C[9] during *PLD* growth of *ZnO*. The charge state of nitrogen vacancies is positive[10] and nitrogen vacancies can diffuse and drift in Si_3N_4.[11] In summary, during *PLD*, properties of Si_3N_4 insulator can be changed due to the formation of additional mobile defects in Si_3N_4, e.g. positive nitrogen vacancies which can diffuse to compensate for concentration inhomogeneities in the Si_3N_4 layer and which can drift in an electric field to the ZnO/Si_3N_4 interface and the $Si_3N_4/p\text{-}Si$ interface if a negative bias (Figure 1(**a**)) and a positive bias (Figure 1(**b**)) is applied to the *Au* top electrode, respectively. This redistribution of defects in leaky Si_3N_4 causes the observed increase in low-frequency capacitance which is much larger than the expected saturation capacitance of non-leaky Si_3N_4. We have modelled the increased saturation capacitance of the leaky Si_3N_4 insulator by the small *ac* signal component resistance R_{ZI} at ZnO/Si_3N_4 interface and by the small *ac* signal component resistance R_{IS} at $Si_3N_4/p\text{-}Si$ interface. Because both small *ac* signal resistances R_{ZI} and R_{IS} are caused by the mobile defects in leaky Si_3N_4, we assume that R_{ZI} and R_{IS} are equal. The R_{ZI} or R_{IS} parameters describe the *ac* resistance of Si_3N_4 and have been added to the equivalent circuit model to describe the low frequency capacitance, mainly below 5 kHz. Only by adding R_{ZI} or R_{IS} to the *RC* elements, the frequency dependent capacitance could be modelled in the whole frequency measurement range. It should be noted that the *ZnO* and *p-Si* have cut off frequencies above 50 kHz. R_{ZI} and R_{IS} are least when Si_3N_4 insulatorSi_3N_4 insulator becomes most leaky if *ZnMnO* with 2 at. % *Mn* and if *ZnCoO* with 2 at. % *Co* are deposited on $Si_3N_4/p\text{-}Si$ under small and under large oxygen partial pressure ($Zn_{0.98}Mn_{0.02}O\_LP$, $Zn_{0.98}Mn_{0.02}O\_HP$, $Zn_{0.98}Co_{0.02}O\_LP$, $Zn_{0.98}Co_{0.02}O\_HP$, Table S6). Because of the *PLD* growth of *ZnO* under different conditions, different mobile defects are formed in the Si_3N_4. Therefore, the modelled R_{ZI} or R_{IS} parameters show a huge variation, namely from 15 mohm-m^2 ($Zn_{0.98}Co_{0.02}O\_HP$, *A1* contact) to 300000 mohm-m^2 (*ZnO_HP*, *A1* contact). By dividing the resistance R_{ZI} or R_{IS} by the thickness of Si_3N_4 (Table S1) one obtains the *DC* resistivity of Si_3N_4 in the range from 1.7×10^5 ohm-m ($Zn_{0.98}Co_{0.02}O\_HP$, *A1* contact) to 3.7×10^9 ohm-m (*ZnO_HP*, *A1* contact). The resistivity of Si_3N_4 in $Zn_{0.98}Co_{0.02}O\_HP$ at 55.7 MV/m (Table S6) is in agreement with the resistivity of Si_3N_4 thin films (5×10^5 ohm-m) which has been reported by Kobayashi et al.[12] who measured a conduction current density of 10^{-4} A/m^2 at an electric field of 50 MV/m. (s.a. Figure 2**b** in K. Kobayashi et al.[12] (0.5 MV/cm and 10^{-8} A/cm^2))
By taking minimum depletion capacitance C_{dep} value as difference between capacitance at low frequency and high frequency in strong inversion, doping concentration N_a of *p-Si* have been calculated from the measured frequency dependent capacitance curve of reference sample.

C_{dep} is considered as a space charge capacitance (C_{SC}) to calculate maximum depletion width (X_d) of the *p-Si* is as follows.

$$X_d = \frac{\varepsilon}{C_{SC}}, \tag{S2}$$

where ε is static dielectric constant. We obtained N_a (Eq. S3) and barrier height (ϕ_b) (Eq. S4) of p-Si with the X_d from equation S2 as follows:

$$N_a = \frac{4V_t * ln(\frac{N_a}{n_i})\varepsilon}{eX_d^2}, \tag{S3}$$

$$\phi_b = \frac{eN_aX_d^2}{4\varepsilon}, \tag{S4}$$

where e electron charge, n_i is intrinsic carrier concentration and $V_t = \frac{KT}{e}$. From above calculation we obtain N_a as 2.431×10^{22} m^{-3} and bulk potential for *p-Si* as 0.730 V.

$$V_{bi} = \frac{eN_aX_d^2}{2\varepsilon} \tag{S5}$$

$$\phi_b(p-Si/Au) = V_{bi} - V_t * ln(\frac{N_a}{n_i}) \quad (S6)$$

The Schottky barrier height $\phi_b(p-Si/Au)$ between *p-Si* and *Au* has been calculated with the depletion approximation by taking the modelled capacitance of bottom contact (C_{st}) as C_{SC} in (Eq. S2) to calculate the depletion width of the *p-Si* (X_d) at *p-Si/Au* interface. X_d and N_a of *p-Si* (2.431E+22 m^{-3}) is used to calculate the built in potential V_{bi} (Eq. S5). The calculation of $\phi_b(p-Si/Au)$ (0.34 eV) from Eq. S6 agrees with difference of work function of Au (4.80 eV)[13] and work function of Si (5.07 eV)[14].

The cut off frequencies of *RC* elements describing the individual regions are listed in Table S7. Top contact (*TC*) and bottom contact (*BC*) at the interfaces of *Al/ZnO* and *p-Si/Au* respectively form a Schottky barrier. The cut off frequencies of contacts are below 220 kHz. Modelled cut off frequency (8.8 MHz) of mobile surface state defects from Si_3N_4/*p-Si* interface is same for all samples (8.8 MHz) and cut off frequencies of static surface state defects lie between 100 kHz and 950 kHz. Mobile interface defects in *ZnO* have larger cut off frequencies (MHz) than static defects (kHz).

References

1. He, G., Sun, Z., Liu, M. & Zhang, L. *Scaling and Limitation of Si-Based CMOS* (John Wiley & Sons, Ltd, 2012). https://onlinelibrary.wiley.com/doi/pdf/10.1002/9783527646340.ch1.
2. Ou, S.-L., Liu, H.-R., Wang, S.-Y. & Wuu, D.-S. Co-doped zno dilute magnetic semiconductor thin films by pulsed laser deposition: excellent transmittance, low resistivity and high mobility. *J. Alloy. Compd.* **663**, 107–115 (2016).
3. Udayakumar, S., Renuka, V. & Kavitha, K. Structural, optical and thermal studies of cobalt doped hexagonal zno by simple chemical precipitation method. *J. Chem. Pharm. Res* **4**, 1271–1280 (2012).
4. Vegesna, S. V. *et al.* Tunable large field magnetoconductance of zno, znmno, and zncoo thin films. *J. Appl. Phys.* **125**, 215305 (2019).
5. You, T. *et al.* An energy-efficient, BiFeO3-coated capacitive switch with integrated memory and demodulation functions. *Adv. Electron. Mater.* **2**, 1500352, DOI: 10.1002/aelm.201500352 (2016).
6. Föll, H. Heterojunctions: Semiconductor technology and nano electronics (Accessed on 07/04/2019).
7. Gonon, P. & Vallée, C. Modeling of nonlinearities in the capacitance-voltage characteristics of high-k metal-insulator-metal capacitors. *Appl. Phys. Lett.* **90**, 142906, DOI: 10.1063/1.2719618 (2007).
8. Khan, I. & Zulfequar, M. Structural and electrical characterization of sintered silicon nitride ceramic. *Mater. Sci. Appl.* **2**, 738–747, DOI: 10.4236/msa.2011.27102 (2011).
9. Kuwabara, A., Matsunaga, K. & Tanaka, I. Lattice dynamics and thermodynamical properties of silicon nitride polymorphs. *Phys. Rev. B* **78**, 064104, DOI: 10.1103/PhysRevB.78.064104 (2008).
10. Di Valentin, C., Palma, G. & Pacchioni, G. Ab initio study of transition levels for intrinsic defects in silicon nitride. *The J. Phys. Chem. C* **115**, 561–569, DOI: 10.1021/jp106756f (2011). https://doi.org/10.1021/jp106756f.
11. Schmidt, H., Gupta, M. & Bruns, M. Nitrogen diffusion in amorphous silicon nitride isotope multilayers probed by neutron reflectometry. *Phys. Rev. Lett.* **96**, 055901, DOI: 10.1103/PhysRevLett.96.055901 (2006).
12. Kobayashi, K., Suzuki, A. & Ishikawa, K. Thermal annealing effect on ultraviolet-light-induced leakage current in low-pressure chemical vapor deposited silicon nitride films. *Thin Solid Films* **550**, 545 – 553, DOI: https://doi.org/10.1016/j.tsf.2013.10.163 (2014).
13. Moll, J. L. *Variable capacitance with large capacity change*, 542–546 (World Scientific, 1991).
14. Pfann, W. G. & Garrett, C. G. B. *Semiconductor Varactors Using Surface Space-Charge Layers*, 547–548 (World Scientific, 1991).
